# Mapping the movie-watching brain with AI-derived semantics

**DOI:** 10.1162/IMAG.a.1300

**Published:** 2026-07-10

**Authors:** Muwei Li

**Affiliations:** Vanderbilt University Institute of Imaging Science, Vanderbilt University Medical Center, Nashville, TN, United States; Department of Radiology and Radiological Sciences, Vanderbilt University Medical Center, Nashville, TN, United States

**Keywords:** fMRI, naturalistic stimuli, LLMs, semantic encoding

## Abstract

Naturalistic paradigms offer a powerful tool to investigate human brain function, but it remains difficult to link rich, continuous movie content to distributed brain activity in an interpretable way. In this study, I use a multimodal large language model (Gemini) as an automated “semantic annotator” to bridge naturalistic movie stimuli, brain responses, and cognitive performance. Using the Human Connectome Project movie-watching dataset, I segmented the film into 293 overlapping clips, prompting Gemini to rate each clip on 11 psychologically interpretable dimensions. Simultaneously, I extracted clip-wise BOLD activation patterns from the fMR images in 360 cortical ROIs. In this way, the AI and the brain effectively “watch” the same movies in parallel. For each brain ROI, I then fit linear regression models to predict clip-to-clip variation in movie-evoked responses from these features. Gemini-derived features robustly predicted movie-evoked responses in temporal, medial parietal, and lateral frontal association cortex, but explained little variance in unimodal somatosensory, dorsal parietal, insular, and piriform regions. Feature-weight maps reflected known functional specializations, and features with the largest global influence overlapped with the most explainable ROIs. Partial least squares analysis revealed that individual differences in resting-state connectivity strength and semantic explainability covaried along an asymmetric intrinsic axis: strongly integrated sensory-opercular systems at rest were associated with poorer AI predictability, whereas a smaller set of dorsal and medial association regions showed enhanced alignment. Finally, regional AI explainability in medial parietal and left perisylvian association areas was positively related to specific cognitive abilities. Together, these findings demonstrate that interpretable features from AI models provide a simple and scalable framework for quantifying AI-derived semantic predictability in naturalistic settings, offering a practical framework for utilizing artificial models as semantic references to probe human neural processing and individual differences.

## 1 Introduction

Over the past decades, cognitive neuroscience has largely relied on highly controlled paradigms using simplified stimuli such as checkerboards, isolated words, or static images to probe the functional specificity of the human brain (Bandettini, 2012; Engel et al., 1997; Gonzalez-Castillo & Bandettini, 2018; Poline & Brett, 2012). While these designs trigger reliable brain responses, they capture only a narrow slice of how the brain operates in a natural context. Over the past decade, however, the field has increasingly turned toward naturalistic paradigms, such as movie watching and narrative understanding, to study the brain in conditions that are closer to real-world experience, where multiple cognitive systems operate together rather than in isolation (Demirtaş et al., 2019; Saarimäki, 2021; Vanderwal et al., 2015). Naturalistic movies elicit robust, distributed, and often highly synchronized BOLD responses across individuals, providing rich opportunities to study large-scale functional organization and intersubject coupling (Kauppi et al., 2010; Meer et al., 2020). However, the “richness” also creates a methodological bottleneck. It presents a continuous, high-dimensional stream of visual, auditory, linguistic, and social information that is difficult to translate into a tractable design matrix (Bordier et al., 2013; Saarimäki, 2021; Simony & Chang, 2020). As a result, linking the rich neural dynamics observed during naturalistic viewing to the content that participants experience remains a major methodological challenge.

Early movie-fMRI work approached this challenge from the sensory end of the spectrum by extracting low-level visual and auditory features, such as luminance statistics, Gabor or wavelet filter responses, and optical-flow-based motion energy, to quantify the stimulus (Nishimoto et al., 2011; Russ & Leopold, 2015). These data-driven features successfully explained responses in the early sensory cortex, but they accounted for much less variance in higher-order regions. This limitation is often referred to as the semantic gap (Hu et al., 2010, 2012; Sonkusare et al., 2019), in which much of the neural activity elicited during naturalistic viewing remains unexplained when using feature spaces grounded only in low-level perceptual information. For example, standard optical-flow metrics can capture that “something moves quickly across the screen” but cannot distinguish whether the motion reflects an angry fight or a joyful dance (Isik & Vessel, 2021; Lefèvre & Baillet, 2009; Russ & Leopold, 2015). Thus, purely low-level feature sets are intrinsically limited in their ability to account for the semantic and social dimensions that are central to naturalistic experience.

In addition to low-level sensory descriptions of the stimulus, some large-scale naturalistic neuroimaging efforts have adopted structured semantic labeling frameworks, such as semantic category labels derived from WordNet, a lexical database widely used in early computer vision and natural language processing research (Huth et al., 2016; Zhang et al., 2020). Compared with motion energy and related sensory features, these WordNet-based annotations provide an explicit, structured vocabulary of objects and actions in the scene (e.g., person, car, walking, speaking). This framework enables more direct comparisons between stimulus content and neural responses in higher-order cortex and has been used to map semantic selectivity during naturalistic movie viewing (Huth et al., 2012). However, despite this progress, WordNet is primarily object- and action-centric, describing what is visibly present but provides only limited access to the richer psychological dimensions that shape human interpretation, such as conversational context, social tension, or narrative structure, and thereby it captures only a subset of the constructs that are likely to drive higher-order cortical responses during movie viewing, leaving many layers of meaning unrepresented (Huth et al., 2012; Thye et al., 2024).

A third line of work leverages deep neural networks trained on visual or audiovisual tasks, using activations from intermediate layers as high-dimensional embeddings of movie content for encoding or decoding models (Rakhimberdina et al., 2021; Sohn et al., 2024; Wen et al., 2018; Yu et al., 2022). These dense embeddings often capture complex structures of the movie, improving prediction in higher-order cortex by representing objects, scenes, and other abstract attributes beyond pixels and motion vectors. Multimodal technique further extends this approach by integrating visual and auditory information to derive even more comprehensive neural representations of naturalistic stimuli (Fu et al., 2025; X. Liu et al., 2025; Nakagi et al., 2024; Villanueva et al., 2025; Zheng & Sun, 2024). However, such models are often treated as “black boxes,” delivering high-dimensional and opaque features in the form of large numerical vectors with hundreds or thousands of values, with no clear meaning attached to each individual dimension. For example, the model may indicate that “dimension 482” or “dimension 917” strongly predicts activity in a specific brain region, yet it remains unclear whether such dimensions represent social interaction, emotional tension, narrative structure, or other latent semantic factors. Current feature-modeling strategies for naturalistic stimuli thus tend to trade-off between interpretability (manual, low-dimensional labels) and completeness (high-dimensional, black-box embeddings), leaving a need for representations that are both rich and human interpretable.

Recent advances in multimodal large language models (LLMs) such as Gemini (https://gemini.google.com) offer a fundamentally new way to address this problem (Gemini Team Google et al., 2025). Trained on vast collections of text, images, and videos, these models develop integrated world models that can infer objects, actions, social relations, emotions, and narrative context directly from raw visual and auditory input. Particularly with recent advances in multimodal LLMs such as Gemini 2.5 Pro, a short movie clip can be translated into detailed, context-sensitive descriptions that resemble human narrative summaries (e.g., “an awkward conversation between coworkers,” “a tense confrontation in a dark hallway”). This has led to the hypothesis that multimodal LLMs can serve as “automatic annotators,” transforming naturalistic stimuli into quantitative feature vectors suitable for regression and encoding. This setup allows for a direct comparison between brain and model, because when a human watches a movie in the scanner and a multimodal LLM “watches” the same movie outside the scanner, the film simultaneously elicits distributed BOLD activity in the brain and structured activation patterns across the internal units within the AI model. If features derived from the model can be mapped onto the functional topography of the brain, this supports a degree of representational alignment between artificial and biological systems. To avoid ambiguity in terminology, I use large language model (LLM) as the general term throughout this work and use multimodal LLM specifically when referring to Gemini’s ability to process visual, auditory, and linguistic information within a unified model interface.

Building on these developments, the present study adopts an interpretable, low-dimensional scoring strategy for movie-fMRI. Using the Gemini model as a multimodal rater, I define a set of 11 psychologically meaningful dimensions, for example, the presence of people, degree of social interaction, threat or conflict, and prompt the model to score each short, overlapping clip (segment) of the continuous movie. This yields a human-interpretable feature space that captures the high-level semantic and social content while remaining computationally tractable. Simultaneously, I extract clip-wise fMRI responses from 360 cortical regions of interest in the Human Connectome Project (HCP) 7T movie dataset (Elam et al., 2021) and fit ROI-wise encoding models that use the Gemini-derived features to predict BOLD activity over time (across clips). The resulting regression coefficients and prediction accuracies (R²) form a semantic map of the movie-watching brain, revealing which regions are most sensitive to each feature and how much variance in their activity can be explained by this AI-derived representation. Because every feature has a clear interpretive label, I can move beyond asking what aspects of the movie each brain region encodes.

While encoding models are commonly evaluated at the group level, I hypothesize that the extent to which an individual brain corresponds to an AI-derived semantic representation may itself be informative. As LLMs are trained on enormous collections of real-world data, they learn common patterns that reflect shared cultural knowledge and broadly experienced patterns of perception, language, and social meaning. In this sense, such models can be viewed as approximating a population-level representational “center”, a distilled abstraction of how information is typically structured and interpreted across many human experiences and learning histories. If so, the degree to which the movie-evoked brain responses of a subject can be predicted from Gemini semantic features provides a measurable index of how closely their neural representations correspond to this inferred normative space. This perspective raises testable questions: Do individuals whose neural responses are more well predicted by the model also demonstrate stronger cognitive performance or more efficient brain communication? To examine this, for each individual, I relate AI-derived semantic predictability accuracy (R²) to two independent indices of individual variation: intrinsic functional connectivity (FC) measured at rest and behavioral performance on higher-order cognitive tasks. I found that variability in semantic explainability covaried with resting-state network strength across the cortex. Furthermore, leveraging the cognitive assessments available in the HCP dataset, I observed that individuals whose movie-evoked activity was better predicted by the semantic model tended to have higher fluid intelligence and related abilities.

Importantly, the term “AI-derived semantic predictability” in this framework requires careful clarification. By quantifying this alignment, I do not imply that the LLM possesses human-like intelligence, consciousness, or biological mechanisms analogous to those of the human brain. Rather, the LLM serves as an artificial semantic reference model derived from large-scale human-generated data. During naturalistic movie watching, brain activity is shaped by fluctuating attention and idiosyncratic mental content. The AI, by contrast, processes the same movie stimulus through a standardized computational procedure and projects it into a simplified, explicit 11-dimensional representational space. In this context, “alignment” refers to the degree of correspondence between neural responses and this artificial semantic reference space. Accordingly, any use of alignment in this manuscript should be read as an operational measure of semantic predictability or correspondence with the explicit AI-derived feature space, rather than as evidence for direct alignment between biological neural activity and the internal hidden representations of the model.

Taken together, this work demonstrates a principled framework for using multimodal large language models to generate interpretable semantic representations of complex naturalistic stimuli and link them to large-scale neural activity and individual cognitive variation. By pairing explicit LLM-derived semantic dimensions with movie-evoked fMRI responses, I show that artificial semantic structure can be mapped onto cortical organization in a transparent and behaviorally meaningful manner. Moreover, the degree to which an individual brain is captured by this AI-based semantic representation reflects both intrinsic network architecture and cognitive ability, suggesting that alignment may serve as a quantitative marker of individual differences in high-level semantic processing. Beyond the specific implementation used here, the framework is extensible. Future studies can design customized semantic features to probe other patterns of brain activity, and test, tune, or refine LLMs and prompting strategies to derive representations that more closely approximate the way the brain itself encodes information. In this sense, the approach establishes a two-way bridge: on one side, it uses AI to help neuroscience by decoding the semantic structure of neural responses under naturalistic experience; on the other side, it uses brain data to calibrate the biological plausibility of advanced AI models. Together, these contributions outline a scalable and interpretable pathway toward studying how meaning is represented in both artificial and biological systems, and how those representational spaces converge or diverge across individuals.

## 2 Methods

### 2.1 Ethics statement

The data involved in this research are publicly available and have been previously approved for use by the Washington University Institutional Review Board. All participants provided written informed consent to participate in this study. The authors did not collect any new data involving human participants.

### 2.2 Participants, imaging, and preprocessing

Imaging data were downloaded from the Human Connectome Project Young Adult (HCP-Y) database (Van Essen et al., 2013). I include all subjects who completed four 7T fMRI scans under the movie-watching task (n = 176), comprising 70 males and 106 females between the ages of 22 and 35 years.

The proposed analyses incorporate structural MRI, resting-state fMRI, and task fMRI (movie watching). The imaging protocol has been described in detail elsewhere (Van Essen et al., 2012). Briefly, 7T fMRI data were acquired using gradient-echo EPI sequences. Each session consisted of scans with opposing phase-encoding directions, with parameters TR = 1,000 ms, TE = 22.2 ms, and 1.6 mm isotropic voxel resolution. T1-weighted structural images (3T) were acquired using a 3D MPRAGE sequence (TR = 2,400 ms, TE = 2.14 ms, 0.7 mm isotropic resolution). A rich set of behavioral measures is available for these participants and was included in the present analyses.

T1-weighted images are nonlinearly registered to MNI space using FNIRT (Jenkinson et al., 2012), and cortical surface reconstructions are generated using FreeSurfer (Dale et al., 1999). These surfaces were then registered to a standard template using MSMAll (Robinson et al., 2018), which aligns cortical areas based on multimodal features rather than geometry alone. Functional preprocessing includes motion correction, distortion correction (Andersson et al., 2003), and registration to the corresponding structural image, followed by nonlinear alignment to MNI space. After spatial normalization, the data were projected to the standard 32k fs LR surface mesh and saved in CIFTI dtseries. Additional preprocessing includes denoising via ICA-FIX (Salimi-Khorshidi et al., 2014), which removes structured noise components while preserving the neural signal, nuisance regression of motion parameters, and high-pass filtering (>0.0005 Hz). For resting-state data, I further performed linear detrending and band-pass filtering (0.01–0.1 Hz).

After preprocessing, regional time series were extracted using the HCP-MMP1.0 multimodal parcellation (Glasser et al., 2016), which defines 360 cortical ROIs (180 per hemisphere) aligned to the same 32k fs LR surface space as the functional data. The atlas was applied directly to the CIFTI dtseries files using HCP workbench software. For each participant and each run, this procedure yielded a 360 × T matrix of ROI-averaged BOLD time series, where T corresponds to the number of volumes in the run. Both resting-state and task-derived regional time series were processed using the same pipeline to ensure consistency across analyses. The resulting regional time series served as the basis for computing functional connectivity, semantic-encoding models, and subsequent individual-differences analyses. To control for the potential confounding effects of subject head motion in downstream analyses, I extracted the run-averaged head motion metric (mean Relative RMS displacement) for each subject directly from the HCP preprocessed motion parameters. I then averaged these values across the four movie runs to derive a single scalar metric of mean head motion for each participant. This metric was subsequently utilized as a strict covariate to rule out motion-induced artifacts.

### 2.3 Movie information and clips generation

Participants completed four movie-watching fMRI runs as part of the HCP 7T protocol: two runs from the CC movie set and two from the HO set. The CC movies consist of short clips drawn from freely available independent films distributed under Creative Commons licensing, whereas the HO movies are composed of professionally edited excerpts from Hollywood films curated and published by a previous study (Cutting et al., 2012). Each run was structured as a compilation of short audiovisual movies that varied widely in emotional tone, narrative structure, visual complexity, and degree of social interaction, providing broad semantic diversity suitable for naturalistic cognitive engagement. Movies were presented sequentially, but each was separated by a 20-second fixation period. These rest intervals temporally isolated consecutive narratives and allowed the hemodynamic response to partially return toward baseline before the next movie began. Participants were given no task instructions other than to watch the movie naturally, and no behavioral responses were recorded. Precise timing information for each movie, including identity, start time, end time, and duration, was obtained from the official HCP metadata and shared through a Supplementary File in this paper.

**Fig. 1.**
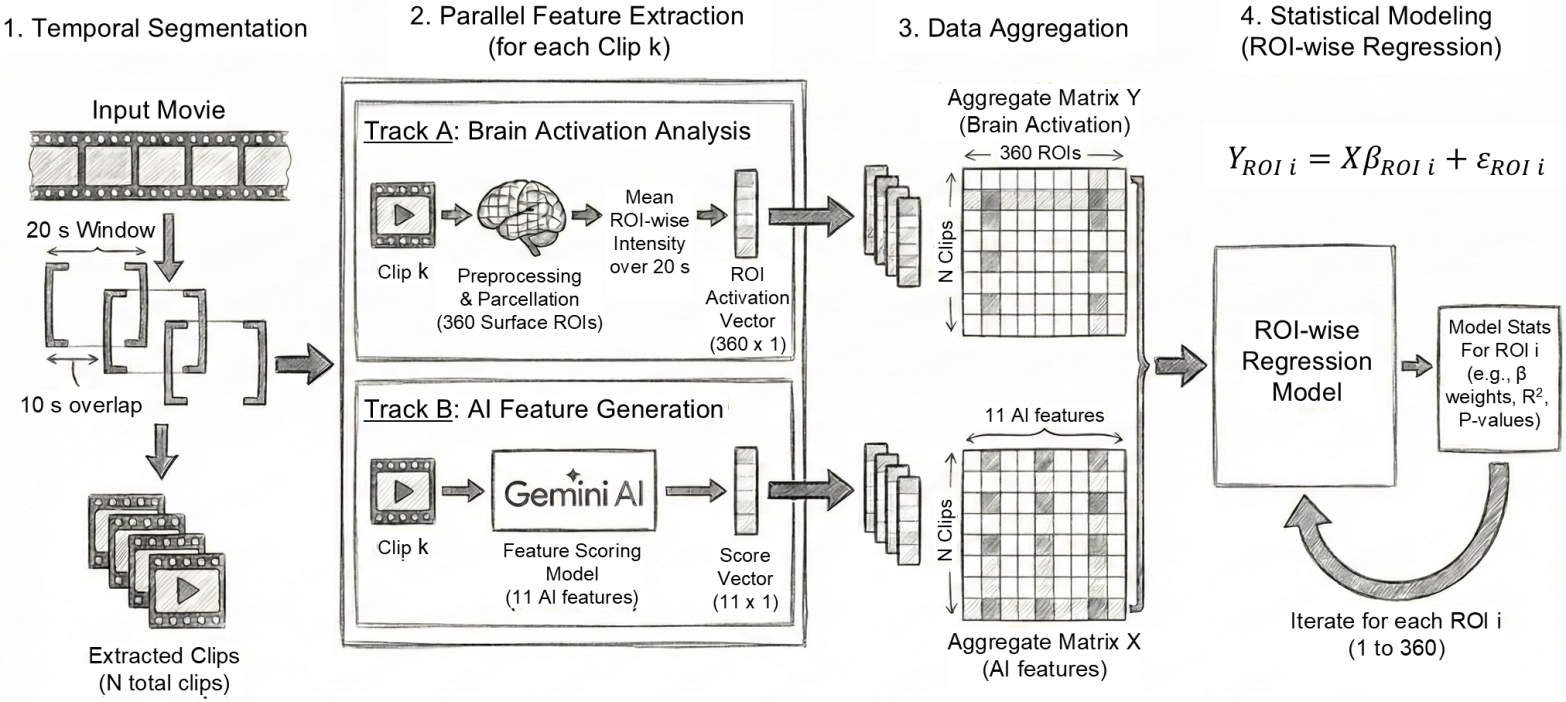
Workflow overview for linking movie-evoked brain activation with AI-derived semantic features. (1) Temporal segmentation. The continuous movie stimulus is segmented into partially overlapping 20-second clips (10-second overlap), producing N clips for analysis. (2) Parallel feature extraction for each clip. Track A: fMRI segment corresponding to each clip undergoes preprocessing and surface-based parcellation into 360 cortical ROIs (HCP-MMP atlas). The mean BOLD intensity over the 20-second window is extracted, yielding a 360 × 1 activation vector. Track B: Each clip is fed into a multimodal Gemini-based model, which outputs an 11-dimensional semantic feature vector describing visual and narrative properties (people presence, scene brightness, valence, etc.). (3) Data aggregation. Across all clips, ROI activation vectors form the matrix Y (N clips × 360 ROIs), and AI-derived feature vectors form matrix X (N clips × 11 features). (4) Statistical modeling: ROI-wise regression. For each ROI (i = 1 … 360), a regression model is fit to quantify how well the 11 AI features explain clip-to-clip variability of activation in each ROI. The model yields regression weights (β), goodness-of-fit (R²), and statistical significance for each ROI.

To support time-resolved semantic annotation and encoding analyses, the continuous movie runs were segmented into shorter overlapping clips. Clip generation was performed using a custom MATLAB script and FFmpeg-based video extraction (https://www.ffmpeg.org). For each of the four movie runs, non-rest intervals were first identified based on the official HCP timing tables. Each continuous movie segment was then partitioned into shorter clips using a sliding-window approach (Fig. 1). Clips were targeted to be 20 seconds in duration to approximate a full cycle of the hemodynamic response, with a minimum allowable length of 16 seconds to accommodate segments near run boundaries. Consecutive clips were generated with 50% temporal overlap (i.e., 10 seconds step size), providing dense temporal coverage of the narrative while preserving continuity across neighboring clips. To account for hemodynamic latency and ensure that the extracted visual content corresponded to BOLD time points used in encoding, a 5-second buffer was reserved at the end of each usable segment. All clips were extracted using FFmpeg, recompressed at a standardized frame rate (24 fps), and saved with synchronized audio to preserve semantic context. The final stimulus set contained 293 clips, each associated with metadata including movie run identity, start time, end time, duration, overlap characteristics, and file name. A manifest table (see Supplementary File for details) documenting all clips and timing information was generated to enable reproducible alignment between video content and fMRI time series.

### 2.4 Clip-wise brain patterns

For each participant, preprocessed fMRI data from the four movie runs were available as ROI-wise time series (M, 360 × T), where rows correspond to MMP ROIs and columns to TRs. Using the same run-specific timing and rest definitions as in the stimulus design, I first identified non-rest frames by excluding all TRs belonging to 20-second fixation intervals within each run. For each run and participant, I then standardized the ROI time series using z-scoring based on non-rest frames only. To reduce global fluctuations, I removed the frame-wise global mean by subtracting the average signal across ROIs at each TR. For each clip defined in the manifest table (293 clips in total), the corresponding time window was shifted forward by 5 seconds to account for the hemodynamic delay between stimulus onset and BOLD response. For a given clip and participant, I extracted the ROI time series within this lagged window and averaged across TRs, yielding a single 360 × 1 vector representing the mean response pattern for that clip in that individual (Fig. 1). To obtain a group-level brain pattern for each clip, I averaged these participant-specific vectors across all subjects. To increase robustness to outliers, I used a 5% trimmed mean across subjects for each ROI (i.e., trimming 5% of the highest and lowest values before averaging). The result is a 360-dimensional group-level response pattern for each of the 293 clips. Importantly, both the group-level clip response patterns and the full set of participant-level clip patterns were stored for downstream analyses, enabling individual-difference analyses.

### 2.5 Clip-wise AI features

Semantic features were extracted from each of the 293 movie clips using a custom automated annotation pipeline built by the Gemini multimodal large language model (Gemini 2.5 Pro, Google DeepMind). A lightweight web-based tool was developed in Google AI Studio to streamline clip processing. Video files were converted to Base64-encoded inline data and submitted programmatically to the Gemini API, which returned structured numerical feature scores. The model was chosen for its native ability to process continuous audiovisual input rather than isolated still frames, allowing it to jointly analyze motion, scene context (captions), facial expressions, dialogue, auditory cues, and temporal dynamics. A zero-shot prompting strategy was used to standardize annotations across clips. The instruction asked the model “to perform a holistic analysis of each clip and assign a continuous score ranging from 0.0 to 1.0 on 11 predefined dimensions” (Fig. 1). The feature set was designed to span a gradient from low-level perceptual properties (i.e., motion intensity, scene brightness) to mid-level social and structural cues (i.e., presence of people, close-up faces, music), and finally to higher-order affective and narrative constructs (i.e., dialogue, social interaction, valence, arousal, threat, and narrative progress). Clips were processed sequentially to comply with API rate limits, using a queued submission system with a fixed 10-second delay between requests. The final dataset consisted of one semantic feature vector per clip, exported as a structured CSV file (see Supplementary File for details) for alignment with brain-pattern data and subsequent encoding analyses. Below is an example of the Gemini output that corresponds to the 366–385 seconds interval of HCP Movie 1. This workflow differed from an interactive chat-based use of the model. All clips were submitted through a programmatically controlled API pipeline, which allowed the prompt, input files, output schema, processing order, and generation settings to be documented consistently across clips. This API-based implementation, therefore, provides a more transparent and auditable annotation procedure than manual prompting through a web chat interface.

- People Presence (1): Human subjects are the primary focus of almost the entire clip. From the opening family portrait to the narrator, the dealership owners, and the archival photographs, people are consistently on screen.
- Faces Closeup (0.4): While there are many people, the framing is largely composed of medium to wide shots (like the group in the showroom or the family sitting). However, the interview shot of the narrator and the framed portraits bring the average up to a moderate–high level.
- Social Interaction (0.6): There is a sense of community and family, but active, on-screen interaction between subjects is limited. Most people are either posing directly for the camera or walking side-by-side, rather than actively conversing with one another.
- Dialogue (1): The clip features a prominent, continuous voiceover narration guiding the viewer through the history of the dealership.
- Music (1): Promotional and historical documentary-style clips of this nature rely heavily on continuous background music to set the tone and drive the pacing.
- Motion Intensity (0.1): The visual pacing is very slow and deliberate. The video relies heavily on static shots, posing subjects, and panning across still archival photographs. The most active movement is a slow walk through the service bay.
- Scene Brightness (0.7): The environments are generally well lit, including the bright, fluorescent-lit modern showroom, the outdoor shots, and the clear, high-contrast black-and-white historical photographs.
- Valence (0.9): The emotional tone is highly positive. It focuses on pride, family legacy, longevity, and community connection.
- Arousal (0.1): The energy level is calm, steady, and nostalgic. It is designed to be informative and reassuring rather than exciting or highly stimulating.
- Threat (0): The content is entirely safe, focusing on a family-owned car dealership. There are no dangerous, aggressive, or suspenseful elements.
- Narrative Progress (0.9): The clip establishes a clear, linear progression, moving from the present day to the founding in 1918, and visually showing the passage of time through the changing cars and generations of owners.

### 2.6 ROI-wise semantic encoding using AI features

To quantify the extent to which AI-derived semantic representations predicted neural responses during movie viewing, I constructed ROI-wise encoding models using the clip-level brain patterns and corresponding Gemini-based semantic scores. For each clip, the previously generated group-level, 360-dimensional brain response vector was paired with its semantic feature vector derived from Gemini scoring. All semantic feature dimensions were standardized (z-scored) across clips prior to model fitting. Figure 1, encoding analyses were performed using ridge regression, implemented separately for each of the 360 cortical ROIs. For each ROI, I examined how well the 11 features could predict BOLD changes across clips. To prevent data leakage and temporal autocorrelation arising from the physical overlap (10 seconds) between adjacent movie clips, I implemented an overlap-purged leave-one-out cross-validation (Purged LOOCV) scheme. In each fold, one clip was held out as the test sample. Crucially, any training clips that originated from the same continuous movie run and shared overlapping timestamps with the test clip were automatically identified and explicitly excluded from the training set. This purging step ensures absolute temporal isolation between the training and testing data. Furthermore, to optimize model generalization and avoid overfitting, I replaced the fixed regularization parameter with a nested grid search within the cross-validation loop to dynamically select the optimal ridge penalty (λ). Prediction performance was measured as the coefficient of determination (R²) between observed and predicted activity across clips. This procedure produced an R² value for each of the 360 ROIs, yielding a spatial map of semantic explainability across the cortex. In addition to prediction accuracy, I quantified feature contribution by examining the magnitude of regression coefficients (β). Moreover, for each feature, I computed the mean absolute coefficient across ROIs as a measure of overall influence, and a second weighted metric in which coefficients were weighted by the ROI-specific R² values, emphasizing features that contributed most to regions with high model predictability.

To map both the global topography of AI-derived semantic predictability and its individual variability, I implemented a strict two-tier modeling architecture.

- Tier 1: Group-Average Encoding (Global Topography). To delineate the general cortical organization of AI-derived semantic representations, the baseline ridge regression encoding models were fit on the group-averaged BOLD signal. Specifically, a 5% trimmed mean across all 176 subjects was computed for each movie clip to attenuate idiosyncratic noise. The global R^2^ prediction map and the specific semantic feature weights were directly derived from this robust, group-level consensus signal.
- Tier 2: Subject-Specific Encoding (Individual Differences). To investigate how intrinsic functional architecture and cognitive traits relate to semantic explainability, the exact same ridge regression pipeline was independently repeated for each of the 176 participants using their individualized BOLD time series. This produced a unique, 360-dimensional R^2^ profile for every subject. Consequently, all subsequent correlational and partial least squares (PLS) analyses linking these individualized R^2^ profiles to intrinsic functional connectivity and cognitive scores were inherently cross-subject, group-level inferences, evaluated for statistical significance using rigorous permutation testing and FDR correction.

### 2.7 Resting-state FC strength and its association with AI-derived semantic-encoding performance

To examine whether intrinsic network organization relates to how well an individual brain aligns with AI-derived semantic features, I quantified resting-state FC strength for each subject and assessed its association with regional encoding performance (R² values from movie-based ridge encoding). Resting-state fMRI data for each participant were processed using the same standardized pipeline applied to the movie runs. Time series were extracted from 360 cortical regions of interest (ROIs) defined by the MMP parcellation. For each subject, I computed a 360 × 360 FC matrix by estimating pair-wise Pearson correlations across ROI time series. The resulting matrices were Fisher-z transformed to stabilize variance. To derive a subject-level measure of intrinsic network organization, I computed a node-wise connectivity strength metric for each region, defined as the mean Fisher-z correlation (negative FCs were set to 0) between that region and all other ROIs. This resulted in a 360-element vector per participant, capturing how strongly each cortical region is functionally integrated with the rest of the cortex during rest.

I next examined whether the spatial pattern of intrinsic FC strength relates to how explainable the activity of each region is by the Gemini-derived semantic features. I employed partial least squares (PLS), a multivariate technique, to statistically assess the correspondence between encoding performance and FC strength. In this analysis, subjects formed the rows of the data matrix (N subjects), and ROIs were the shared variable dimension (360 features). The two input matrices were

X: ROI-wise R² encoding performance (subjects × 360)

Y: ROI-wise resting-state FC strength (subjects × 360).

To ensure that the primary semantic explainability axis linking semantic explainability to intrinsic connectivity was not driven by motion-induced signal degradation, I performed a nuisance regression prior to the PLS modeling. Specifically, I linearly regressed out the subject-level mean head motion parameter from both the 360-dimensional ROI-wise R^2^ profiles and the resting-state FC strength profiles across all subjects. The PLS analysis was then run exclusively on these motion-corrected residual matrices. PLS identifies latent components that maximize the covariance between X and Y. The first latent variable (LV1) was interpreted as the dominant axis linking intrinsic network organization to AI explainability. The statistical significance of LV1 was assessed using permutation testing (1,000 permutations), and the reliability of ROI loadings was evaluated with bootstrap resampling, yielding z-scored stability maps. Regions with stable positive weights on LV1 were interpreted as areas where stronger intrinsic connectivity was systematically associated with higher AI-derived semantic predictability.

### 2.8 ROI-wise association between AI-encoding performance and cognitive scores

To test whether AI-derived semantic predictability relates to individual cognitive abilities, I correlated ROI-wise encoding performance with behavioral measures across subjects. For each participant, I obtained a 360 × 1 vector of encoding performance (R²) from the ridge regression models. These values quantify, for each MMP ROI, how well the movie-evoked responses of that subject are predicted by the Gemini-derived semantic features. For behavior measures, I focused on a set of canonical HCP cognitive indices (age-adjusted), including fluid intelligence (PMAT24), episodic memory (PicSeq), working memory (ListSort), cognitive flexibility (CardSort), inhibitory control (Flanker), age-adjusted fluid cognition composite (CogFluidComp), and crystalized cognition composite (CogCrystalComp). Now I have a subjects × 360 matrix of encoding performance (R²) and a subjects × 7 matrix of cognitive scores, where 7 is the number of available cognitive measures. For each ROI and each cognitive variable, I computed a cross-subject partial Spearman rank correlation between the R² values and the cognitive scores, explicitly controlling for the mean head motion of each subject as a covariate. This produced a 360 × 7 matrix of correlation coefficients (r) and corresponding raw p-values (p). To control for multiple comparisons across all ROI × cognition pairs, I applied a Benjamini–Hochberg false discovery rate (FDR) procedure at q = 0.05, using all correlation p-values pooled into a single vector, and significant effects were defined as FDR-corrected p < 0.05.

## 3 Results

### 3.1 Regional variability in semantic predictability across the cortex

**Fig. 2.**
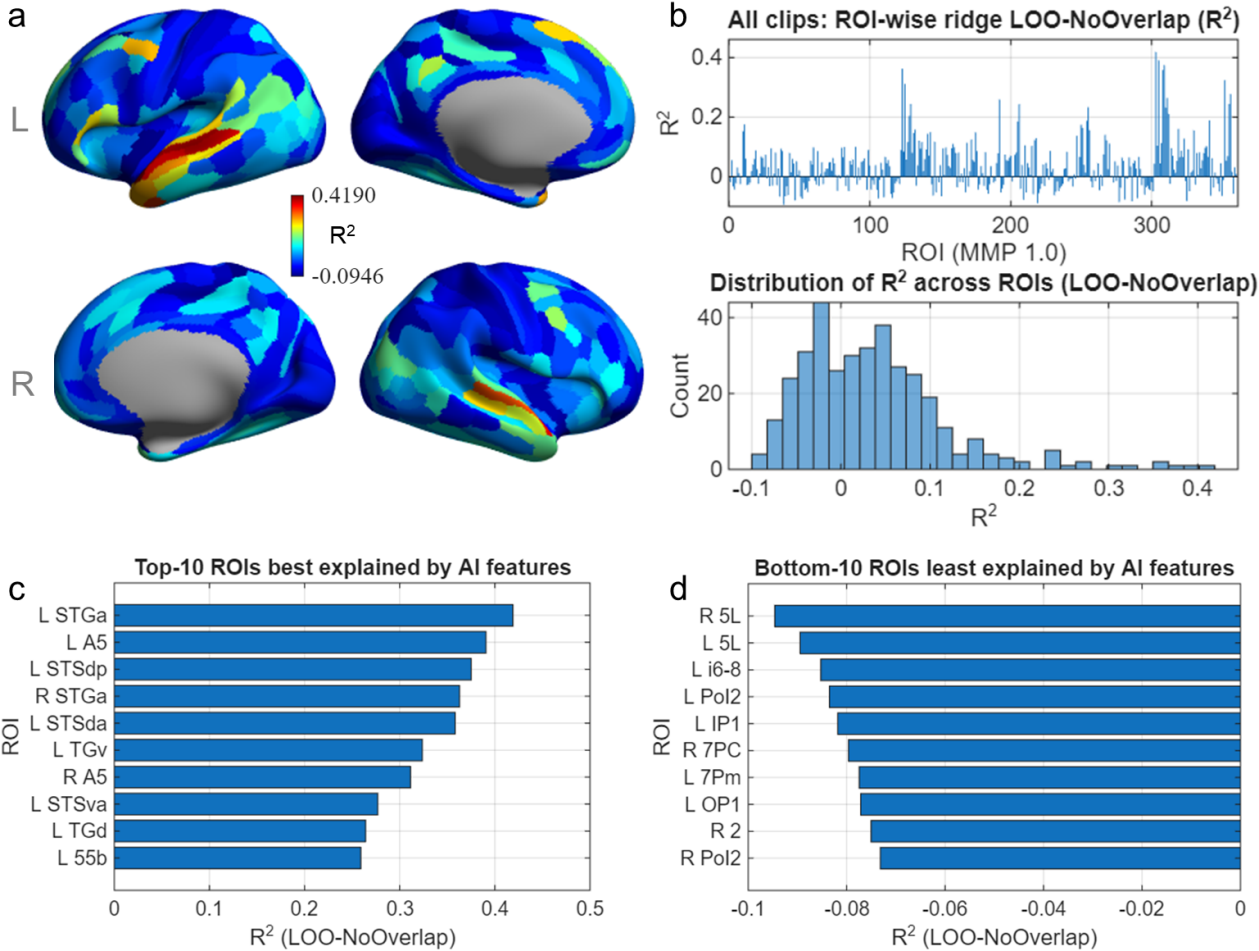
ROI-wise predictive performance of AI features across the cortex. (a) Group-level surface maps of prediction accuracy. Each ROI is colored by its cross-validated coefficient of determination (R²) from the ROI-wise ridge regression model, reflecting how well the 11 AI-derived movie features explain the 20-second windowed brain activation patterns. Warmer colors indicate ROIs whose activity patterns are more strongly predicted by the feature set. (b) Distribution of predictive performance across all ROIs. Top: ROI-wise R² values for all 360 MMP ROIs. Bottom: Histogram summarizing the distribution of R² across the cortex, showing substantial regional variability. (c) Top-10 most predictable ROIs. Bar plot showing the 10 ROIs with the highest cross-validated R² values, indicating regions whose movie-evoked activity is best captured by the AI feature set. (d) Bottom-10 least predictable ROIs. ROIs with the lowest (or slightly negative) R² values, representing regions whose activity patterns are poorly explained by the available AI features.

To determine how well high-level semantic information extracted from the movie clips accounted for regional brain responses, ROI-wise ridge regression models were fit for all 360 cortical ROIs in the MMP atlas. Cross-validated prediction performance was quantified using the coefficient of determination (R²), reflecting how much clip-to-clip variance in movie-evoked activity could be explained by the 11 AI-derived semantic features. As shown in Figure 2a, prediction accuracy varied widely across the cortex, revealing distinct spatial patterns rather than uniform explanatory power. The highest R² values were shown in a distributed set of association regions, including superior temporal sulcus (STS), middle and anterior temporal cortex, precuneus, posterior cingulate cortex, and lateral prefrontal association areas. The distribution of R² values across ROIs (Fig. 2b) further highlights strong regional heterogeneity. While the majority of ROIs exhibited modest but positive prediction values, a subset reached relatively high explanatory performance, exceeding 0.30. A small number of ROIs yielded values near zero or slightly negative, the latter reflecting cases where the cross-validated model performed marginally worse than a mean-only baseline. Ranking the ROIs confirmed these trends. The top-10 most predictable regions (L and R STGa; L and R A5; L STSdp, STSda, and STSva; L TGd and TGv; and L 55b; Fig. 2c) were located almost entirely in the superior temporal gyrus/sulcus and anterior temporal association cortex, with an additional dorsal frontal premotor area (55b) in lateral frontal cortex. Together, these regions belong to networks implicated in higher-order auditory processing, speech and language, and social-narrative understanding. By contrast, the bottom-10 ROIs (R and L 5L; L i6-8; L and R PoI2; L IP1, R 7PC, L 7Pm, L OP1, and R 2; Fig. 2d) included primary and secondary somatosensory areas (2, 5L, OP1), dorsal and inferior parietal association regions (7PC, 7Pm, IP1), a dorsolateral frontal control area (i6-8), and bilateral posterior insular areas (PoI2). These ROIs are mainly associated with somatosensory, sensorimotor, attentional control, or visuospatial processing, and, therefore, show little variance explained by the high-level semantic features modeled here.

As an additional robustness analysis, I also conducted a supplementary Purged Block 10-Fold cross-validation analysis (Supplementary Fig. S1). By dividing the continuous movie runs into 10 contiguous temporal blocks and dynamically tuning the ridge regularization penalty (λ) via a nested grid search, this approach provided a more conservative evaluation with respect to residual temporal dependence. The 10-Fold CV yielded a highly similar spatial topography of semantic predictability and preserved the rank ordering of the top-performing transmodal regions.

### 3.2 Contribution of semantic feature to the prediction model

**Fig. 3.**
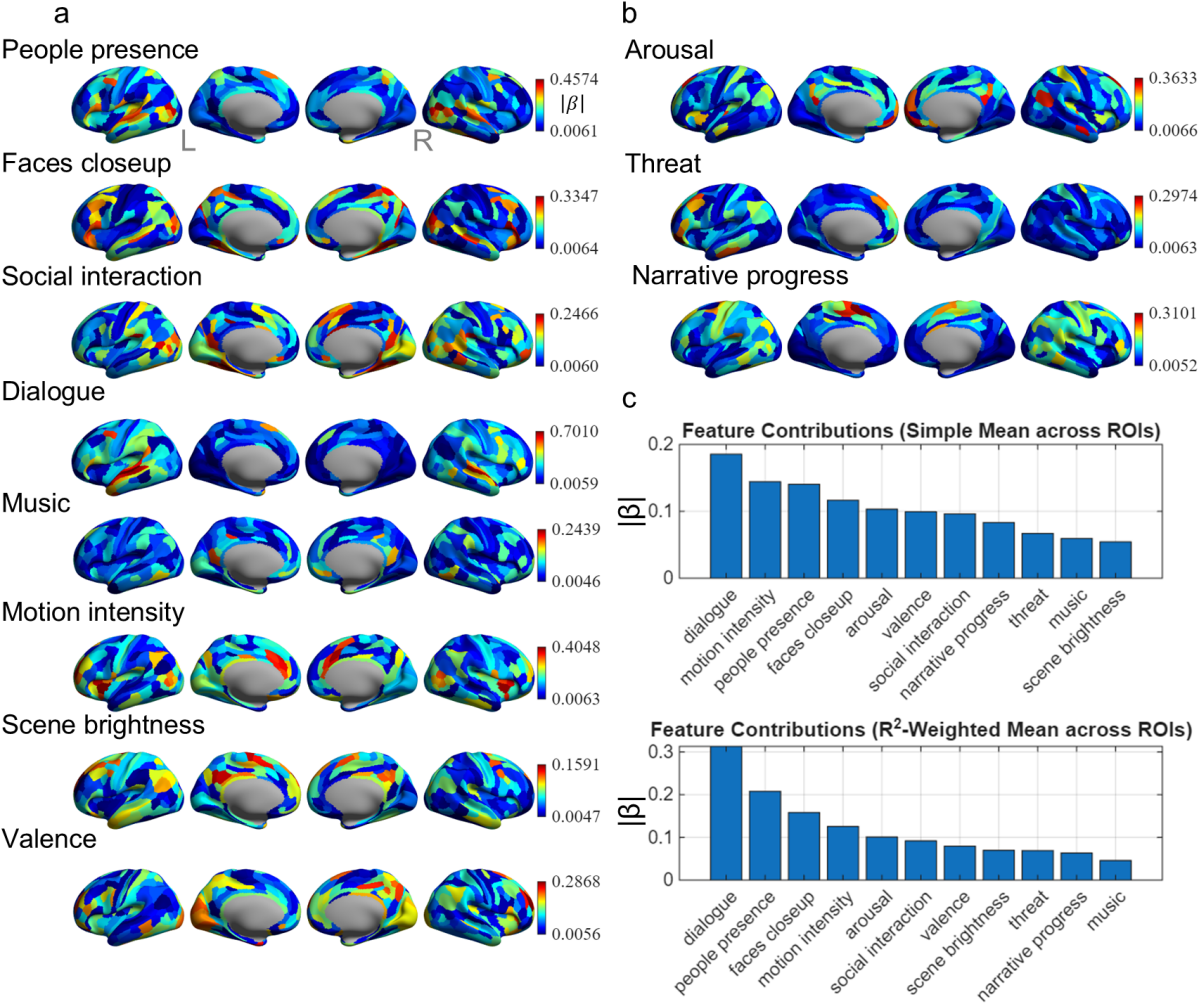
ROI-wise regression weights for individual AI features and their overall contributions. (a) and (b) Cortical maps of regression coefficients (|β|) for 11 semantic-perceptual AI features. Results show the brain-wide spatial distribution of standardized regression weights (absolute value of β) for each feature when predicting ROI-wise activation across all movie clips. Warmer colors indicate stronger associations between a given AI feature and the activation pattern of each cortical region. (c) Feature-level contributions across all ROIs. Top: Mean |β| across 360 ROIs, reflecting the overall influence of each feature on explaining brain activation patterns. Bottom: R²-weighted |β|, which up-weights features that explain ROIs with higher cross-validated predictive accuracy, providing an importance score aligned with model fit.

To characterize how different semantic dimensions from the Gemini model are represented across the cortex, ridge-encoding coefficients were converted to standardized absolute weights (|β|) for each feature and each of the MMP ROIs. The surface maps in Figure 3a and b visualize these |β| values, with higher values indicating stronger sensitivity of the movie-evoked pattern of an ROI to a given feature. The People Presence feature showed its strongest weights in bilateral TPOJ3 (temporo-parieto-occipital junction, multimodal association cortex) and LO3 (lateral occipital visual association cortex), as well as the right TF (ventral temporal fusiform cortex). These areas lie at the interface of occipital, temporal, and parietal regions and are commonly implicated in high-level visual analysis of people and objects, consistent with a sensitivity to the presence of humans in complex scenes. The Faces Close-Up feature exhibited pronounced weights in right area 44 (inferior frontal cortex) and a set of bilateral dorsal/posterior visual regions, including V6A, VMV1, and VMV2. These areas span frontal regions involved in orofacial and speech-related motor representations and medial/ventral occipital regions associated with detailed visual analysis. Social interaction weights are concentrated in bilateral 31a (posterior cingulate–retrosplenial default-mode area) and bilateral parahippocampal regions PHA2 and PHA3. This pattern aligns with a medial temporal–posterior cingulate network involved in contextual, episodic, and social-scene processing. The dialogue feature showed the largest single coefficients across the entire matrix in left A5 and bilateral STGa, closely followed by high weights in right A5 and left STSdp. These regions are located in the superior temporal gyrus and sulcus and adjacent belt areas and are classically associated with higher-order auditory and speech processing. For music, the strongest coefficients appeared in bilateral TA2 (auditory association cortex), left 31a (posterior cingulate), left POS1 (parieto-occipital sulcus), and right s32 (ventromedial prefrontal cortex), consistent with a distributed network spanning auditory, medial parietal, and medial prefrontal regions engaged by musical and affective context. Several features recruited more frontally distributed systems. Motion intensity showed its largest weights in bilateral MI (middle insular cortex) and bilateral frontal opercular regions FOP4, together with the right dorsal medial frontal area p32pr. This pattern corresponds to cingulo-opercular and insular control networks often linked to salience and integrated sensory-motor processing, indicating that perceived motion intensity in the clips is encoded in multimodal control and interoceptive hubs rather than early visual cortex alone. Scene brightness weights were concentrated in left medial frontal and parietal midline regions, including 9p, 7m, 6mp, 31pv, and 31pd, which overlap with default-mode territories along medial prefrontal and posterior cingulate cortex. Affective and appraisal-related features showed distinct prefrontal-limbic signatures. Valence assigned its highest weights to a highly specific set of fronto-limbic hubs, peaking exclusively in right 9-46d and right 46 (dorsolateral prefrontal regions), left 47m (orbitofrontal cortex), left PeEc (perirhinal–ectorhinal cortex in the medial temporal lobe), and right 23c (posterior cingulate). This combination of dorsolateral, orbitofrontal, and medial temporal areas is consistent with networks involved in evaluating the emotional meaning and value of complex events. Arousal weights were maximal in right 10v (ventral anterior prefrontal area), right 8Ad, and bilateral 10r, along with strong contributions from right STSva, indicating a ventromedial and dorsal frontal network, plus superior temporal association cortex. Threat weights localized primarily to a distinct set of left orbitofrontal and lateral prefrontal ROIs, most notably a47r, 8Av, 10pp, 8BL, and p47r, regions associated with evaluation of potential risk, punishment, and behavioral control. Finally, narrative progress was most strongly expressed in a distinct set of cingulo-opercular and dorsal frontal eye-field regions. Its highest weights mapped directly onto left SCEF, left 24dd, bilateral 6mp, and left FEF, consistent with a network supporting sustained control, monitoring, and alignment of attention as the story advances.

Panel 3c summarizes the overall contribution of each feature by averaging |β| across all ROIs (and weighting by ROI-wise prediction performance). Dialogue showed the largest overall contribution to explaining cortical patterns, followed by People Presence and Motion Intensity, with Faces Close-Up, Arousal, and Valence contributing at intermediate levels and Music, Threat, Narrative Progress, and Scene Brightness contributing more modestly. Together, these results indicate that the AI-derived semantic space is not uniformly expressed across the cortex. Features tied to speech, social agents, and dynamic action account for a substantial portion of explainable variance, whereas features such as brightness or low-level soundtrack properties play a comparatively smaller role in shaping the distributed movie-evoked patterns in this dataset.

### 3.3 Semantic explainability axis linking AI-derived predictability to intrinsic connectivity

**Fig. 4.**
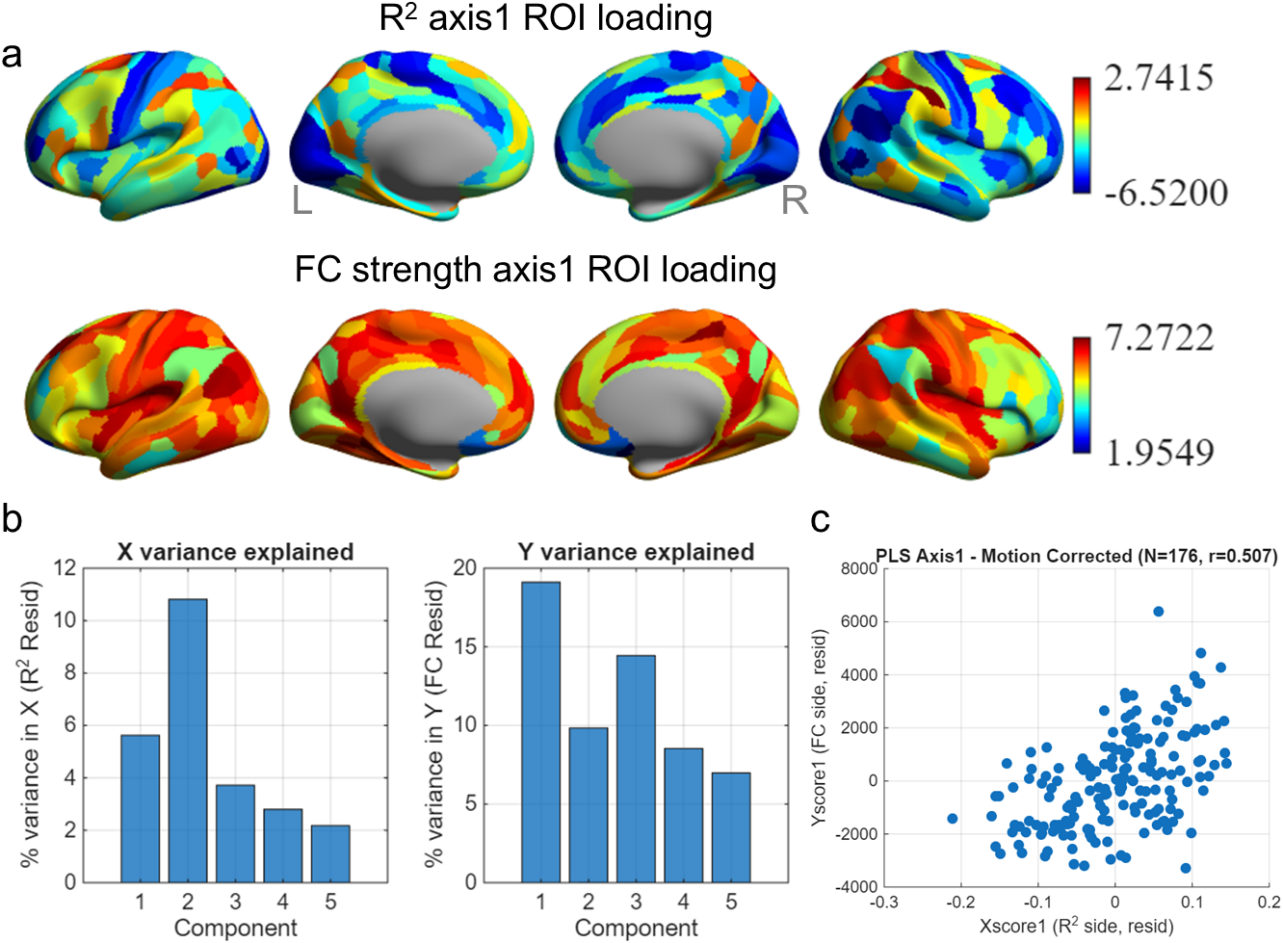
Partial least squares (PLS) analysis linking ROI-wise R² profiles and rsFC-strength profiles across subjects. (a) PLS Axis 1 ROI loadings. Top row: spatial loading pattern for the first latent variable (Axis 1) derived from the across-subject R² profiles (how well AI features explain clip-wise brain activation patterns of each subject). Bottom row: corresponding loading pattern for rsFC-strength profiles. Warm colors indicate ROIs with strongly positive loadings; cool colors indicate strongly negative loadings. These loadings reflect ROIs that contribute most to the covariation between R² and FC-strength patterns across subjects. (b) Variance explained by PLS components. Left: percentage of variance in the R² side (X) explained by each PLS component. Right: percentage of variance in the FC-strength side (Y) explained by each component. Component 1 explains the largest share of variance on both sides, indicating the dominant cross-subject mode of R²-FC covariation. (c) Scatterplot of X-scores (R² side) versus Y-scores (FC-strength side) for all subjects.

To relate individual differences in AI explainability to intrinsic functional architecture, I applied partial least squares (PLS) to two ROI × subject matrices: (i) semantic explainability (R²) from the encoding models and (ii) resting-state FC strength for the same MMP ROIs. The first latent variable (PLS Axis 1) captured the dominant mode of covariation between these two profiles across participants (Fig. 4). At the subject level, X-scores (R² side) and Y-scores (FC-strength side) were positively correlated (r = 0.52; Fig. 4c), indicating that individuals whose cortical activity patterns were better predicted by the AI-derived semantic features tended to occupy a similar position along an intrinsic connectivity gradient. Axis 1 also explained the largest share of variance in both spaces (Fig. 4b), making it the primary “AI-derived semantic predictability” dimension examined here.

The spatial loading patterns for Axis 1 revealed a striking asymmetry between the two modalities (Fig. 4a). On the R² side, loadings were largely shifted toward negative values (range: −6.5 to +2.7, median: –2.6), with relatively few ROIs showing strongly positive contributions. The most positive R² loadings were predominantly found in bilateral dorsal and lateral association cortex, including inferior parietal area PFt, anterior intraparietal area (AIP), medial intraparietal area (MIP), and 7PL, as well as premotor area 6a. In these regions, subjects with stronger resting FC along Axis 1 also tended to show higher AI explainability, reflecting a modest “aligned” mode where a more strongly connected dorsal attention, parietal–cingulate network accompanies better capture of movie-evoked responses by the semantic model. In contrast, many sensory and sensorimotor regions exhibited strongly negative R² loadings while simultaneously showing high positive FC-strength loadings on the same axis. This was particularly evident in early visual and motion-sensitive cortex (MST, V4t, V3, MT, V2, V1, FFC), dorsal mid-cingulate area 24dv, somatomotor regions (areas 3a, 1, 5m), and insular/opercular ROIs (e.g., FOP4, PoI1/2). For these ROIs, individuals with stronger intrinsic connectivity along Axis 1 tended to show lower semantic explainability, yielding a pronounced “anti-aligned” component of the solution. The FC-strength loadings themselves were almost uniformly positive (range: 2.0 to 7.3, median: 5.9), with peaks in medial temporal (L PHA1), inferior parietal (L PGi), dorsal prefrontal (L 8Ad), and posterior insular/opercular regions (L PoI2, L OP2-3), as well as ventromedial visual cortex (L VMV2).

### 3.4 AI-derived semantic explainability predicts individual differences in cognitive performance

**Fig. 5.**
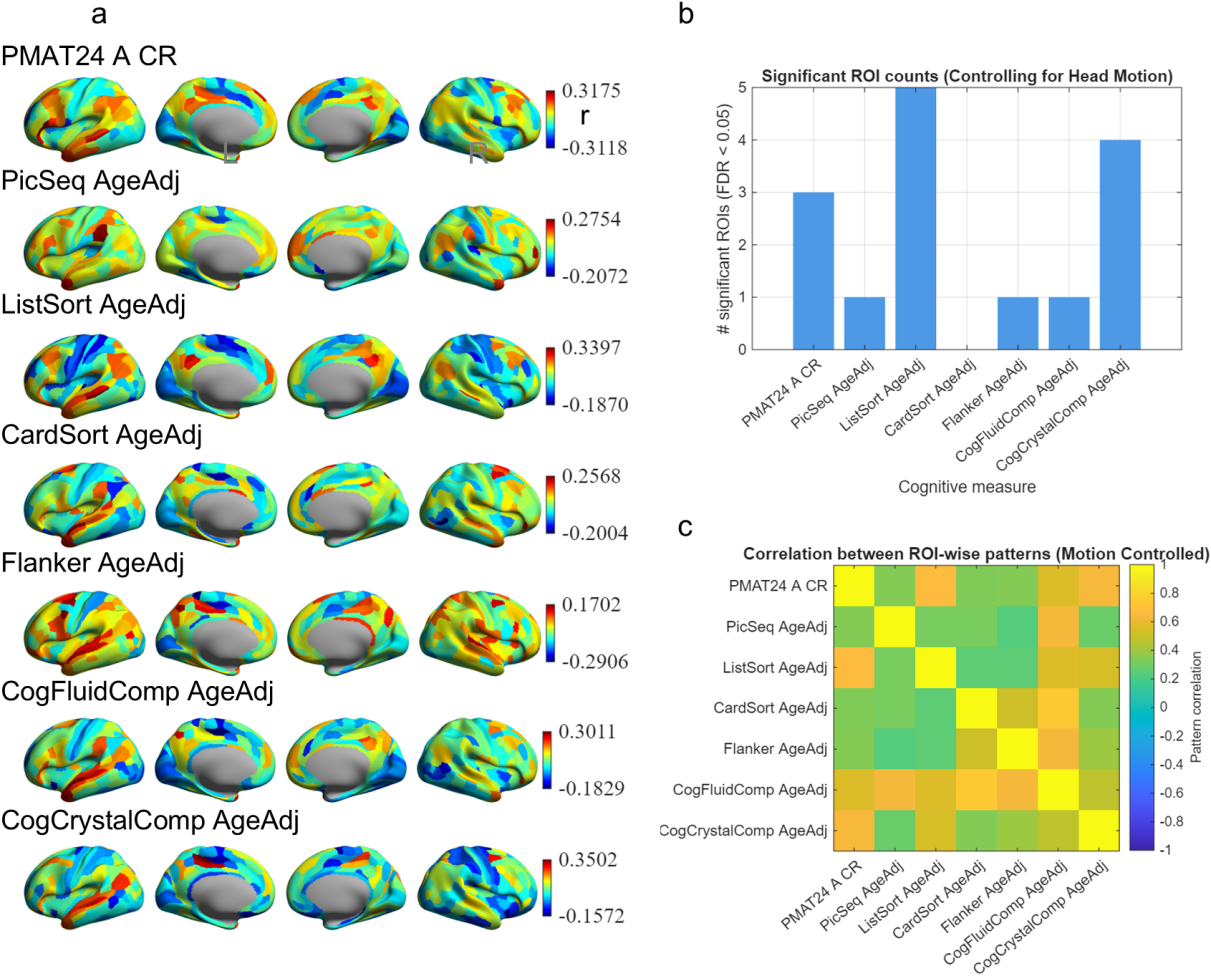
Associations between ROI-wise explainability (R²) and individual cognitive abilities. (a) ROI-wise Spearman correlations between explainability and cognition. Surface maps show the resulting r values for each ROI (MMP parcellation). Warm colors denote positive associations (higher cognitive score associated with higher explainability), while cool colors denote negative associations. (b) Number of significant ROIs for each cognitive measure. For each cognitive score, the number of ROIs showing a significant correlation with R² (FDR < 0.05 across all ROI × cognitive tests) is plotted. (c) Similarity between explainability–cognition spatial patterns. A correlation matrix showing pair-wise pattern similarity between the ROI-wise correlation maps of all cognitive measures. Each entry reflects the Pearson correlation between two r-maps from panel (a).

I next examined whether individual differences in AI-derived semantic predictability were related to cognitive performance (Fig. 5). For each subject and each ROI, I correlated the movie-encoding R² with seven HCP cognitive scores. After FDR correction (q < 0.05) across all ROI-cognition tests, significant associations emerged in specific, highly localized networks (Fig. 5b). Fluid intelligence (PMAT24) was significantly positively associated with explainability in left area 45 (inferior frontal gyrus/Broca’s area) and left STSvp (superior temporal sulcus), while showing a negative association with left FOP1 (frontal operculum). Working memory (ListSort) performance showed strong positive associations with explainability in the posterior cingulate and superior temporal cortex, specifically bilateral area 31pd, bilateral STSvp, and left STSdp. For Inhibitory Control (Flanker), I observed a single significant negative association in left area 5L (somatosensory cortex). Episodic memory (PicSeq) also revealed a significant positive association in the left parietal region PF. The Fluid Cognition Composite (CogFluidComp) showed its only significant peak in left STSda (anterior superior temporal sulcus). Finally, the Crystallized Cognition Composite (CogCrystalComp) showed robust effects, with the strongest positive correlations found in left medial parietal regions L 5mv and L 23c, left STSdp, as well as right FEF (frontal eye field). Together, these results reveal a consistent topography in which individuals whose neural responses in medial parietal, posterior cingulate, and lateral temporal language/social association areas are better predicted by the AI semantic features tend to demonstrate superior cognitive performance.

Despite the sparsity of FDR-significant ROIs, the spatial patterns of semantic explainability–cognition coupling were systematically related (Fig. 5c). The ROI-wise r maps for fluid measures (PMAT24, ListSort, CogFluidComp) were strongly correlated with each other, sharing a medial parietal and superior temporal profile. These results suggest that individuals whose cortical activity, particularly in medial parietal, posterior cingulate, and temporal association regions, is better captured by the Gemini-derived semantic features tend to show stronger performance on higher-order cognitive tests.

## 4 Discussion

In this study, I used a multimodal large language model as an automated “semantic annotator” to link naturalistic movie content with large-scale patterns of human brain activity. By prompting Gemini to generate a compact set of perceptual, social-affective, and narrative features for hundreds of overlapping clips from HCP 7T movies, I showed that these AI-derived descriptors robustly explained time-resolved cortical responses across a broad association network, while leaving many primary sensorimotor and insular regions largely unexplained. The feature-weight maps revealed distinct topographies for different dimensions of AI features. At the individual level, a multivariate analysis revealed a pronounced inverse relationship in the unimodal cortex. Subjects whose resting-state networks were more strongly functionally integrated tended to show lower AI-derived semantic predictability in primary sensory, motor, and insular regions, even as alignment remained concentrated in higher-order association cortex. Finally, individual differences in explainability within specific ROIs were selectively related to fluid and crystallized cognitive abilities. Together, these findings provide an initial demonstration that LLM-based video annotations can serve as a powerful, scalable bridge between the semantic structure of complex real-world stimuli, intrinsic brain network organization, and variation in cognitive performance across individuals.

### 4.1 AI-derived semantic predictability concentrated in the association cortex

A first key insight from these analyses is that AI-derived semantic predictability is highly non-uniform across the cortex, and it closely follows known functional hierarchies. The LLM-derived feature space yielded the highest explainability in transmodal association regions along the superior temporal, anterior temporal, medial parietal, and lateral frontal cortices, whereas primary sensory, somatomotor, insular, and piriform areas were only weakly predicted. Consistent with this pattern, the association ROIs with the highest R² carried substantial weights for the most influential features, particularly dialogue, people presence, and motion intensity, indicating that naturalistic speech, social content, and dynamic visual events are the main drivers of movie-evoked responses in these regions. Rather than being tied to a single feature, however, these high-R² hubs showed coordinated contributions from multiple narrative and social-affective dimensions, consistent with the idea that association cortex supports flexible cognition by combining many types of information within the same regions (Fusi et al., 2016; Rigotti et al., 2013). By contrast, the poor predictability in somatosensory, motor, insular, and olfactory cortices likely reflects, first, the fact that the HCP movie paradigm provides relatively weak and indirect stimulation of these systems, for example, very limited actual bodily sensation, interoceptive change, or olfactory input, so that there is little reliable variance for any semantic model to capture. A second, complementary possibility is that the current 11-dimensional feature set may under-represent the specific low-level and bodily codes that these regions carry, which are not explicitly modeled by the LLM prompt. Together, this divergence highlights both the strength and the limitation of using a compact, interpretable AI feature space. It captures the dominant high-level psychological drivers of movie-evoked activity in transmodal networks but likely misses aspects of low-level and bodily processing that may only emerge under richer task manipulations and with more specialized feature sets in future work.

This interpretation should be constrained to the explicit semantic feature representation used here. Prior neuro-AI studies often compare layer-wise embeddings from open models with different levels of cortical processing, with earlier layers sometimes better capturing lower-level sensory regions and later layers better capturing association cortex. Because Gemini is a closed-source system accessed through an API, its internal hidden states and layer-wise embeddings are not available for analysis. Therefore, the weak prediction observed in primary sensory regions should not be interpreted as a general limitation of multimodal LLM representations themselves, but rather as a limitation of the compact, high-level, prompt-derived semantic features examined in the present study.

While the highest predictive performance was concentrated in temporal and lateral frontal regions, focusing only on global peaks may obscure the regional contribution patterns of the LLM-derived abstract features. To further characterize how these high-level semantic dimensions relate to lower-level linguistic cues, I examined the feature-encoding profiles (absolute β weights) across distinct cortical nodes (Supplementary Fig. S2). This localized analysis revealed regionally differentiated weighting patterns. Although auditory and attention-associated regions (e.g., L STGa and R TPOJ3) were dominated by speech and human presence, other higher-order regions showed stronger weighting for abstract semantic dimensions. For instance, in the right VMV3, a high-level visual association area, the largest weight shifted from human presence to social interaction, consistent with sensitivity to broader social-context information in the scene. Most notably, in dorsolateral prefrontal cortex (L 8Av), a region associated with higher-order evaluation, the LLM-derived threat feature showed the largest absolute weight. Together, these regional patterns suggest that, although basic linguistic features are prominent in specific sensory-associative cortices, LLM-derived continuous psychological dimensions may provide additional information for characterizing the distributed networks engaged during naturalistic viewing.

One potential concern is that the robust cortical predictions might simply be driven by one or two dominant features rather than the full richness of the semantic space. To address this possibility, a principal component analysis (PCA) was performed on the AI-derived annotations. Iteratively reconstructing the encoding models using an increasing number of principal components revealed that predictive accuracy in the top-performing regions was not dominated by the first one or two components alone. Instead, encoding performance increased substantially, primarily when higher-order principal components were integrated into the model (Supplementary Fig. S3). This confirms that the robust predictive accuracy observed in transmodal association cortices is driven by a high-dimensional, nuanced combination of abstract semantic features rather than a few simple dominant signals.

### 4.2 Intrinsic network architecture shapes AI-derived semantic predictability

The PLS analysis shows that individual differences in AI-derived semantic predictability reflect not only how the brain responds during movie viewing, but also how its intrinsic functional networks are organized at rest. Resting-state FC is widely used to characterize the baseline organization of functional connections across cortical regions (Buckner et al., 2013), and here the first PLS axis captured a dominant semantic explainability dimension: the degree to which an individual brain approximates the AI-defined semantic space during naturalistic viewing is systematically constrained by its resting-state network organization. Importantly, however, the ROI-level pattern of PLS weights indicates that this axis does not simply reflect a uniform “more connectivity, more explainability” relationship. Axis 1 is dominated by a large set of visual, motion-sensitive, somatosensory, and insular/opercular regions that show strongly positive weights for FC strength but robustly negative weights for AI explainability. In other words, individuals with more strongly integrated unimodal networks at rest tend to show less of their movie-evoked variance in these regions captured by the Gemini-derived features. In contrast, a smaller cluster of dorsal parietal and medial posterior association regions (including superior parietal and posterior cingulate/medial parietal ROIs) shows a more classical positive-positive pattern, where greater intrinsic connectivity strength goes along with higher semantic predictability. Thus, the axis is highly asymmetric, and this opposing pattern between unimodal and association regions closely follows well-established macroscale gradients from sensorimotor to transmodal cortex, where unimodal systems at one end carry concrete sensory and bodily codes, while default-mode and higher association networks at the other end support abstract, integrative representations (Knodt et al., 2023; Sydnor et al., 2021; Vogel et al., 2024). One possible explanation is that in individuals whose intrinsic architecture places more “weight” on sensory and opercular systems, movie-evoked responses in those regions may be dominated by low-level signals that fall outside the current 11-dimensional semantic feature set, producing strong FC but weak AI explainability. Conversely, individuals nearer the transmodal end of the axis may express more stable, high-dimensional mixed representations in dorsal and medial association cortex that align more directly with the AI feature space, while unimodal regions play a relatively smaller role in the shared variance captured by the model. Together, these findings highlight that resting-state network architecture selectively shapes where in the cortical hierarchy semantic predictability emerges. Future work could build on this by decomposing intrinsic connectivity within and between specific networks, examining dynamic connectivity states during naturalistic viewing, and testing whether similar asymmetric patterns arise when semantic features are expanded to better cover low-level and bodily dimensions.

### 4.3 AI-derived semantic explainability in association cortex relates to cognitive ability

The associations between ROI-wise explainability and cognition indicate that AI-derived semantic predictability is not only a descriptive property of movie-evoked responses but also carries behavioral relevance. Although the number of FDR-significant ROIs was modest, the effects were spatially specific and converged on a coherent network profile. Across all measures, individuals whose neural responses in medial parietal and left-lateralized language–social association areas were better predicted by the AI semantic features tended to demonstrate superior performance on higher-order cognitive tests. This topography aligns well with prior work implicating medial parietal–posterior cingulate cortex and lateral temporal–parietal association areas in semantic cognition, memory, and complex reasoning (Binder et al., 2009; Buckner & Carroll, 2007; Crittenden et al., 2015). The observation that explainability in these regions relates to both fluid and crystallized cognition suggests that what matters for cognitive performance is not simply how strongly they activate, but how efficiently their movie-evoked patterns can be captured by a compact, semantically interpretable feature space. In other words, individuals whose association areas express neural responses that are “well behaved” with respect to the AI semantic axes tend to perform better on demanding reasoning, working memory, and vocabulary tasks.

Importantly, this specific form of AI-derived semantic predictability should be distinguished from a more general neural typicality effect. Because the naturalistic stimulus and the AI-derived feature matrix are the same for all subjects, one possibility is that the AI-encoding model may partially reflect similarity to the empirical group-average response (i.e., how far a subject deviates from the mean) (Jamison et al., 2025; Y. Liu et al., 2025). To assess this possibility, I conducted a supplementary control analysis comparing the cognitive associations of AI-based encoding (R^2^_AI_) with those of each individual’s temporal alignment to the actual group-average BOLD signal (R^2^_normative_). As shown in Supplementary Figure S4, alignment with the group average was associated with widespread positive correlations with cognitive scores across many cortical ROIs, consistent with a broad neural typicality effect. In contrast, AI-derived semantic encoding showed a much sparser and more regionally specific topography concentrated primarily in transmodal association regions. These contrasting patterns suggest that the 11-dimensional LLM feature space is unlikely to be explained solely as a proxy for a global population template. Instead, it may provide a more selective semantic reference for identifying regions in which semantic-model correspondence is associated with higher-order cognition.

These findings are consistent with the “AI as center” hypothesis I proposed. Large multimodal models such as Gemini are trained to approximate a distilled abstraction of how information is typically structured and interpreted across many human experiences (Gemini Team Google et al., 2024; Wang et al., 2024). Therefore, AI-derived semantic predictability, operationalized as R² for movie encoding, indexes how closely neural representations during naturalistic viewing of an individual approach this inferred normative semantic space. Notably, an AI is hard to read the human mind from fMRI alone, but watching the same movie gives both systems a common semantic anchor, much like providing a test with its answer key, making it possible to quantify how the representations from each individual correspond to or diverge from normative patterns.

In addition, the pattern is neither global nor uniformly positive. For the Flanker inhibitory-control task, the correlations between explainability and performance were modest overall but skewed toward negative values (roughly −0.29 to 0.17), indicating that better inhibitory control tended to be associated with slightly lower AI-based predictability, especially outside the association hubs highlighted by the other cognitive measures. Within the present framework, this should not be viewed as a “worse” brain state. Instead, it could suggest that the neural patterns supporting strong inhibition are less directly expressed along the compact semantic space defined by the Gemini features and are, therefore, harder for this particular model to capture. Given the small effect sizes and sparse pattern, we treat this as a tentative observation and place greater emphasis on the more robust positive associations.

Finally, the overall patterns of correlation maps for fluid measures (PMAT24, ListSort, CogFluidComp) are highly similar and share a medial parietal profile, indicating that the effects concentrate in a consistent, theoretically meaningful network. This suggests that AI-derived semantic predictability is most behaviorally relevant when it occurs in specific association hubs, rather than being uniformly high across the cortex. Future work with larger and more diverse samples could test the robustness and generality of these associations, examine longitudinal links between alignment and cognitive change, and extend the framework to other domains by tailoring the AI feature space. In this way, R²–cognition relationships provide not only a behavioral validation of the AI-derived semantic representation, but also a potential quantitative marker of how efficiently key cortical hubs implement a normative, center-like organization of meaning.

### 4.4 LLM-based annotations as an artificial semantic reference for naturalistic stimuli

More precisely, the annotations in this study should be treated as an artificial semantic reference for naturalistic stimuli, not as a validated substitute for human semantic processing. The value of the LLM-derived feature space lies in its scalability, consistency, and interpretability, while its validity must be evaluated through converging evidence such as human-rating comparisons and neural predictive performance.

Beyond the specific findings, this work illustrates a general strategy for leveraging large multimodal models to probe human cognitive processing. Rather than treating AI as a “black box” that produces high-dimensional, opaque embeddings, I used it to generate a small set of explicit features, positioning foundation models as flexible “annotation engines” that expose their internal semantic structure in a form that is both neurobiologically meaningful and statistically tractable. In this sense, the AI model provides a population-level representational template, while the brain data reveal where and how individual nervous systems approximate, diverge from, or reorganize around that template.

Meanwhile, it is important to acknowledge that the present 11-dimensional feature space is necessarily incomplete. Many aspects of movie experience, fine-grained visual form, detailed motor plans, interoceptive and visceral states, and long-range narrative dependencies are not fully captured by these few semantic axes. Moreover, the neural patterns I model are derived from clip-wise BOLD changes using a sliding-window scheme that is closely related to the dynamic-variation approaches often applied to resting-state data (Chen et al., 2015; X. Liu et al., 2018; Sun et al., 2023). This representation is well suited to continuous movie stimulation, which, like rest, lacks clear trial boundaries and unfolds over long time scales, but it inevitably compresses the underlying dynamics and may miss faster or more complex BOLD fluctuations that are only partially coupled to the current semantic features. Future work could combine LLM-based annotations with richer models of brain dynamics to better capture the full temporal structure of movie-evoked activity.

A further important limitation of utilizing LLMs for psychological annotation is the absence of a traditional human-rated reference standard and the potential variability of generative foundation models (Abdurahman et al., 2024; Cui et al., 2025; Demszky et al., 2023). Because LLM outputs can vary depending on underlying API updates and sampling parameters, it is reasonable to question whether the AI-derived semantic time series are reliable or instead reflect stochastic variability. To address annotation stability, I evaluated the reproducibility of the annotations through a perturbation-based stress test. I performed an independent replication run 4 months after the initial extraction, deliberately introducing contextual perturbation by prompting the model to evaluate a much denser 30-dimensional semantic space. Despite these perturbations and the time interval, the original 11 core features isolated from this perturbed run showed substantial temporal stability (average Pearson r = 0.815; Supplementary Fig. S5).

While generating a dense multi-dimensional human reference standard across the entire continuous movie dataset is highly susceptible to rater fatigue and inter-rater variability, I conducted a targeted manual annotation experiment on a representative subset of 30 video clips to directly compare human and AI performance. Behaviorally, the automated pipeline demonstrated strong construct validity by exhibiting robust positive correlations with human judgments across the feature space, successfully capturing both explicit visual properties and highly subjective semantic dimensions such as narrative progress and threat (Supplementary Fig. S6). Crucially, when evaluated for predictive validity using the cross-validated encoding framework, the AI-derived features achieved cortical predictive performance highly comparable with the manual human annotations. In fact, the automated model demonstrated distinct predictive advantages over human raters, specifically within transmodal cortical regions associated with higher-order semantic processing and social cognition. The fact that these AI-derived features align closely with human perception while selectively capturing unique clip-to-clip BOLD variance suggests that the zero-shot prompting framework yields structured and informative annotations rather than unstructured noise. Together, the observed annotation stability, behavioral construct validity, and neural predictive relevance support the use of multimodal LLMs as a scalable and empirically constrained psychological annotation tool.

Despite these limitations, the framework is highly flexible. Because the features are defined by simple natural-language prompts, it is straightforward to modify or extend the semantic space for different study designs. Researchers can easily re-prompt the model to emphasize other constructs (e.g., threat types, social hierarchy, or clinically based behaviors) or expand the feature set when richer coverage is needed, much like constructing task-specific but interpretable embeddings for different cognitive domains. To empirically examine the scalability of this framework, I conducted a supplementary analysis by expanding the semantic space from the original 11 dimensions to a broader set of 30 features (Supplementary Fig. S7). This expanded set incorporated additional high-level cognitive, affective, and narrative dimensions, such as “Theory of Mind,” “Social Hierarchy,” and “Surprise.” When evaluated under the overlap-purged cross-validation scheme, the 30-dimensional model extended the spatial extent of predictability into broader cortical networks. This supplementary analysis suggests that the proposed framework is not limited to a small, predefined annotation set; rather, researchers may be able to extend LLM prompting to extract higher-dimensional, customized semantic features, enabling richer artificial representational spaces to be examined in relation to human cortical topography. A practical advantage of this pipeline is that it is technically lightweight and accessible, even for investigators without a software engineering background. Using Google AI Studio and the Gemini API, semantic scoring of long videos can be implemented with only a few lines of short, human-readable prompts, with the platform handling video ingestion, multimodal processing, and model hosting behind the scenes. This lowers the barrier for clinical and translational researchers who may not have the resources to build computer-vision models, but who routinely collect rich behavioral, neuroimaging, or symptom data that could benefit from standardized, scalable annotation of complex stimuli. To explicitly test whether these abstract dimensions contribute distinct explanatory variance beyond basic sensory properties, I evaluated the collinearity and independent predictive power of a fully expanded 30-dimensional feature space (Supplementary Fig. S8). Because real-world cinematic features naturally covary, a pair-wise correlation analysis confirmed substantial shared variance among certain explicit and abstract dimensions. To isolate the unique neural contributions of the higher-order semantics, I partitioned the expanded space into a 15-feature low-level sensory baseline model and a 15-feature abstract semantic model. The resulting whole-brain encoding maps demonstrated a striking spatial divergence. The low-level baseline model successfully explained variance up to an amount of 0.3238, with its predictive power highly concentrated within early visual and auditory cortices. Conversely, the abstract feature model yielded an exceptional predictive performance peaking at an out-of-sample of 0.2046. Crucially, these abstract dimensions exhibited a unique cortical topography that stood in sharp contrast to the sensory model by showing a pronounced preference for transmodal association networks and language hubs. This distinct spatial dissociation empirically confirms that the high-level semantic dimensions capture a substantial and spatially segregated component of neural variance that standard low-level detectors cannot access.

The 30-feature extension also clarifies both the benefit and the limitation of expanding the semantic space. The improved prediction in occipital and visual-association regions likely reflects the inclusion of visual properties such as color saturation, visual clutter, scene complexity, and motion-related descriptors that were absent or only coarsely represented in the original 11-feature model. At the same time, some regions that were strongly predicted by the original feature set, including primary auditory cortex, showed reduced performance after expansion. This decrease may reflect two complementary factors. First, the 30-feature annotations were generated in an independent extraction session using a newer, faster Gemini model, introducing some annotation variance relative to the original Gemini 2.5 Pro scoring. Second, adding many features that are weakly relevant to auditory cortex can dilute the contribution of the core auditory and dialogue-related predictors under ridge regression, because the L2 penalty distributes shrinkage across the full coefficient set. Thus, the expanded model demonstrates the scalability and customizability of the framework, but it also shows that larger prompt-defined feature spaces should be tuned to the neural system and research question under study.

A further important direction is to test whether similar annotation and encoding maps can be reproduced with independent open-weight multimodal systems, such as Qwen-VL or related model families. I did not treat such systems as direct substitutes in the present analysis because many currently available open-weight video pipelines emphasize the visual stream and often require separate audio or speech-recognition modules to handle dialogue and sound. For naturalistic movie stimuli, this separation may weaken the joint audiovisual integration needed to rate high-level constructs such as threat, social conflict, or narrative progress. Nevertheless, future work should explicitly compare natively multimodal commercial APIs with open-weight visual-language and audio-language pipelines to determine which findings are model specific and which reflect more general properties of AI-derived semantic annotation.

Looking ahead, this AI-assisted semantic annotation framework suggests a two-way relationship rather than a one-directional borrowing of AI models to explain neural data. On the one hand, LLM models offer a powerful toolset for cognitive and clinical neuroscience, providing high-coverage, customizable descriptors of naturalistic stimuli and candidate representational spaces for interpreting brain activity. On the other hand, the brain can serve as a rich source of constraints and validation signals for improving AI itself, especially in domains where current models still struggle: long-time scale integration, high-order abstraction, and durable memory (Lake & Baroni, 2023; Parisi et al., 2019; Pulvermüller et al., 2021). For example, default-mode and medial temporal networks integrate emotional and narrative information over tens of minutes during movies, linking the affective tone of a scene to events that occurred many scenes earlier. Embedding such brain-inspired temporal and hierarchical principles into future multimodal models could help them move beyond local, short-context descriptions toward a deeper understanding of extended real-world events. In this view, interpretable, LLM-based annotations are not only a convenient tool for studying the brain but also a stepping stone toward a tighter, mutually informative dialogue between biological and artificial intelligence.

## 5 Conclusions

This study demonstrates that a large multimodal language model can serve as a highly scalable, synthetic semantic baseline to link naturalistic movie content, brain activity, and individual cognition. Gemini-derived semantic features robustly predicted movie-evoked responses in temporal and medial parietal association cortex, while largely failing to explain unimodal and insular regions, and individual differences in resting-state network organization selectively shaped where this AI-derived semantic predictability emerged. Explainability in medial parietal and left perisylvian association areas was further related to fluid and crystallized cognitive abilities, indicating that correspondence with an AI-derived semantic space in specific hubs is behaviorally meaningful. Although the current 11-dimensional feature set is incomplete, its prompt-based, easily customizable design offers a flexible and accessible framework that future work can extend to richer features, additional brain systems, and tighter, bidirectional links between biological and artificial intelligence.

## Supplementary Material

Code Source Files Videos

Supplementary Figures

## Data Availability

The MRI data used in this study are available in the HCP database https://www.humanconnectome.org/. To ensure full computational reproducibility, the complete suite of custom MATLAB scripts in this study is provided as a comprehensive supplementary archive accompanying this manuscript. They can also be found at https://github.com/geyerou/Brain-AI-Alignment. Software and toolboxes that are used in this study: The Gemini-based feature scoring tool is available upon request from the author. Please provide the email address associated with your own Google Gemini account. Gemini: https://gemini.google.com/ Google AI Studio: https://aistudio.google.com/ FFmpeg: https://www.ffmpeg.org/ CIFTI: https://www.nitrc.org/projects/cifti/ GIFTI: https://www.nitrc.org/projects/gifti/ HCP workbench: https://www.humanconnectome.org/software/connectome-workbench
