# Supplementary figures and images for: Mapping the movie-watching brain with AI-derived semantics
Source: Imaging Neurosci (Camb). 2026 Jul 10;4:IMAG.a.1300. doi: 10.1162/IMAG.a.1300 (PMC13358718; doi:10.1162/IMAG.a.1300)

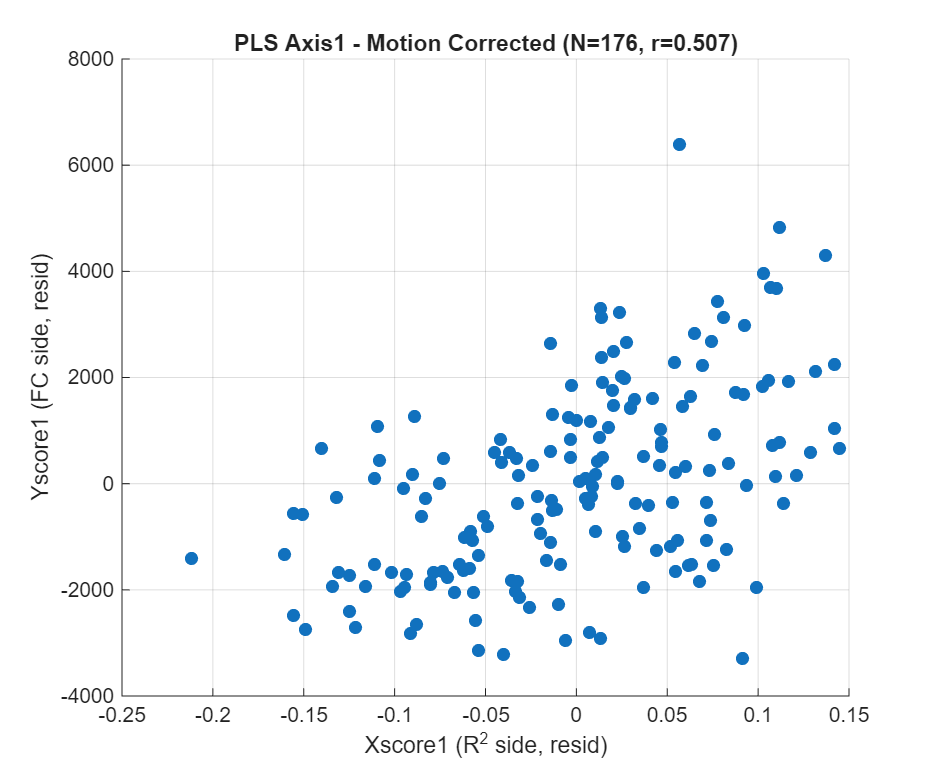

Supplement: Code Source Files Videos [file IMAG.a.1300_supp_Code_SourceFiles_Videos.zip › Supplementary/SourceData/Figure4/PLS_axis1_subject_scores_motion_corrected.png]

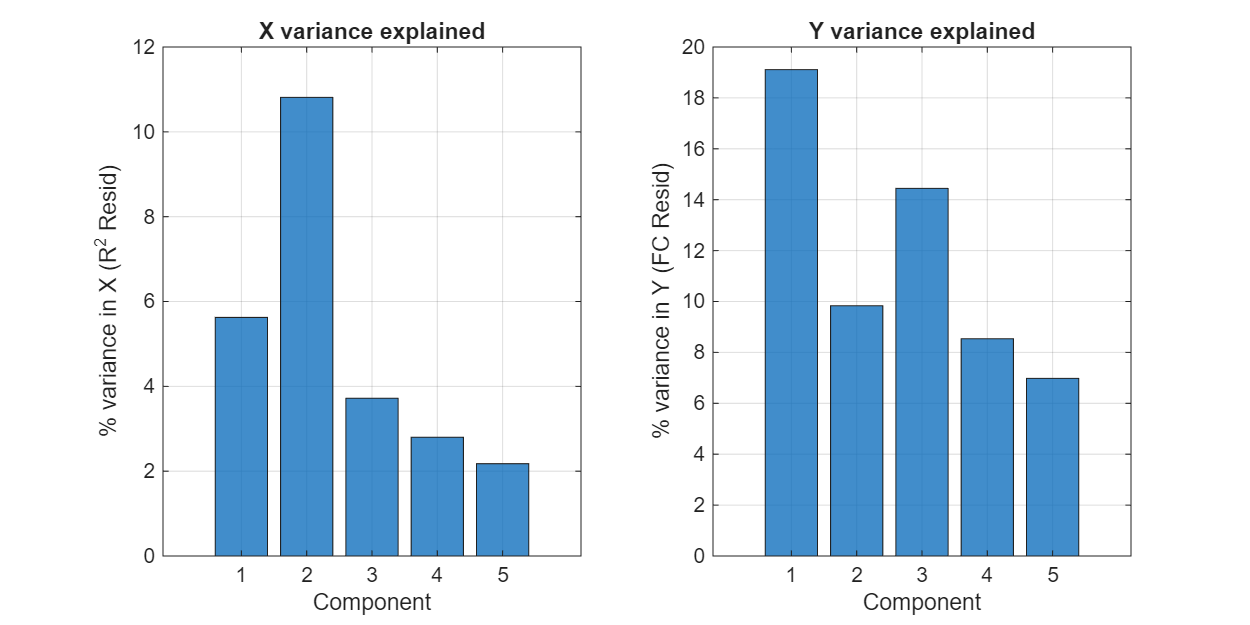

Supplement: Code Source Files Videos [file IMAG.a.1300_supp_Code_SourceFiles_Videos.zip › Supplementary/SourceData/Figure4/PLS_PCTVAR_motion_corrected.png]
