# Supplementary material for: Mapping the movie-watching brain with AI-derived semantics
Source: Imaging Neurosci (Camb). 2026 Jul 10;4:IMAG.a.1300. doi: 10.1162/IMAG.a.1300 (PMC13358718; doi:10.1162/IMAG.a.1300)
Supplement: Supplementary Figures [file IMAG.a.1300_supp_Figures.pdf]

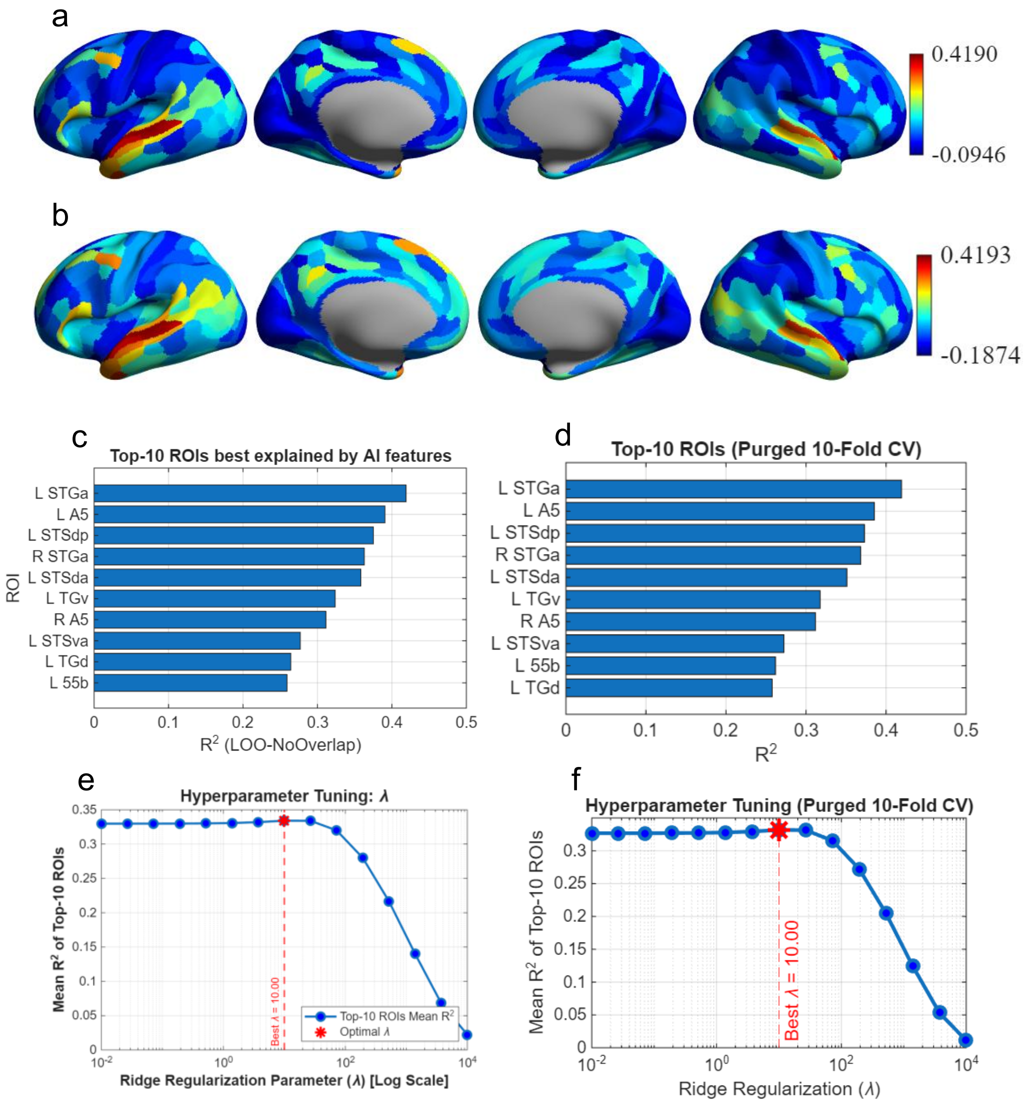

Figure S1. Robustness of AI-brain encoding performance across different cross-validation strategies. (a) Group-level surface maps of prediction accuracy (R^2) generated using the Purged Leave-One-Out Cross-Validation (LOOCV) scheme, which is used in the main manuscript to maximize data efficiency. (b) Cortical maps generated using a Purged Block 10-Fold CV, showing a nearly identical spatial distribution of semantic predictability. (c) The Top-10 most predictable cortical parcels (ROIs) derived from the Purged LOOCV model. (d) The corresponding Top-10 ROIs derived from the Purged Block 10-Fold CV model. (e) Nested hyperparameter tuning curve for the Purged LOOCV. A logarithmic grid search identified the optimal ridge regularization parameter that maximized out-of-sample prediction accuracy for the top-performing association regions. (f) Nested hyperparameter tuning curve for the Purged 10-Fold CV.

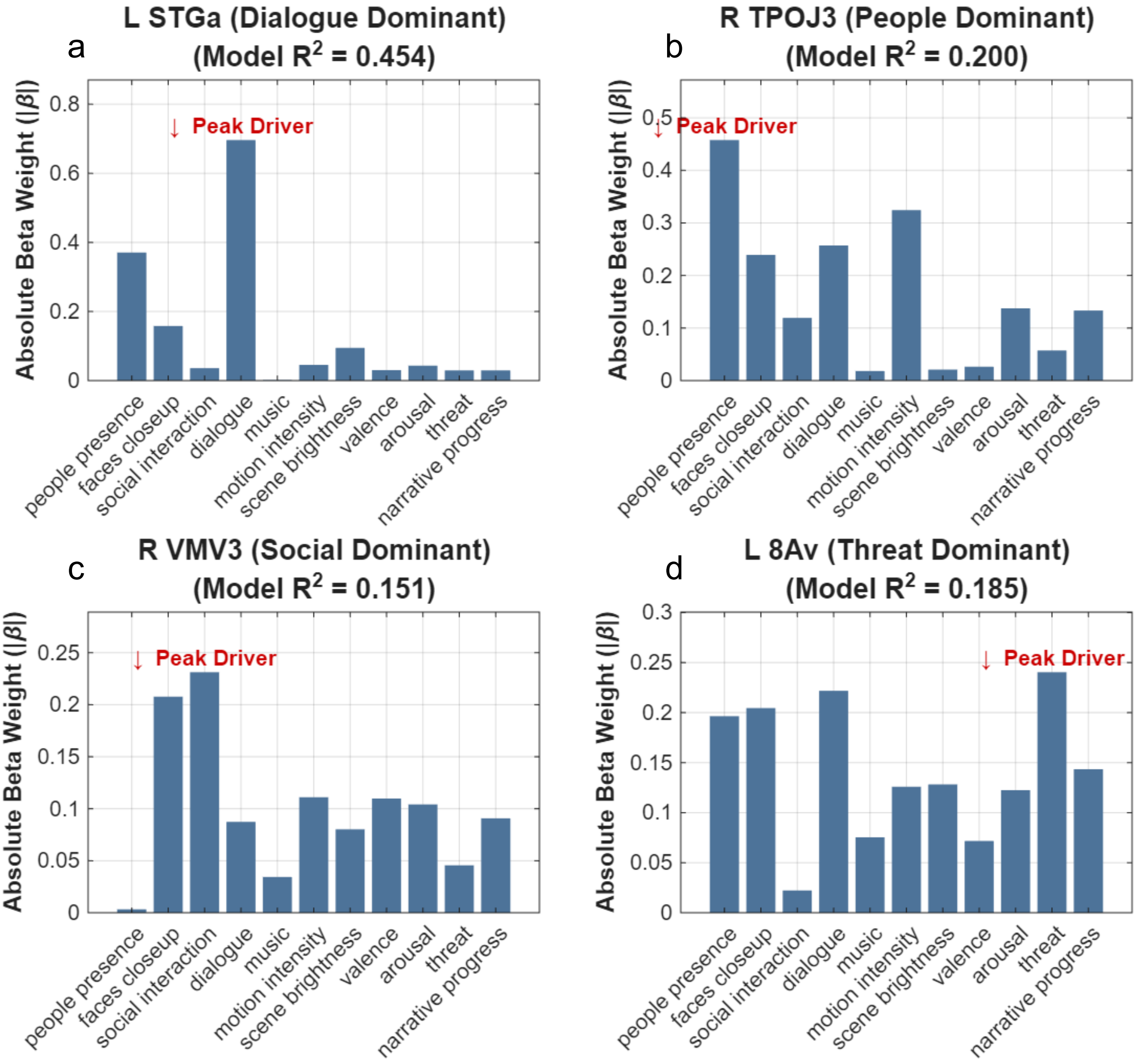

Figure S2: Feature Encoding Profiles Across Representative Cortical Nodes. Bar charts display the absolute beta weights derived from an 11-feature Ridge regression encoding model during naturalistic movie watching. Four representative regions of interest (ROIs) with robust out-of-sample prediction accuracy ($R^2 > 0.1$) are shown. The ROIs are (a) L STGa, (b) R TPOJ3, (c) R VMV3, and (d) L 8Av.

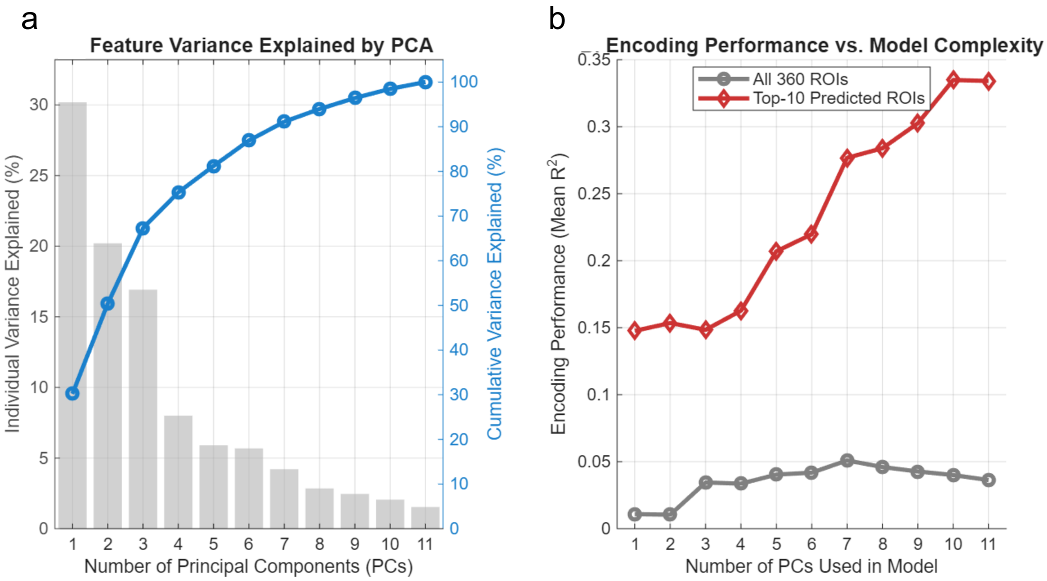

Figure S3. Spatial specificity and dimensional requirements of AI-derived semantic spaces in predicting cortical activity. (a) Feature variance explained by Principal Component Analysis (PCA). The gray bar chart displays the individual percentage of variance explained by each of the 11 derived semantic components across the 293 naturalistic movie clips. The blue line and circular markers indicate the cumulative variance explained, demonstrating that the semantic profile is distributed across a multidimensional space rather than being concentrated within one or two dominant components. (b) Out-of-sample encoding performance as a function of model complexity. The line plots demonstrate the out-of-sample prediction accuracy (mean R^2 under the overlap-purged leave-one-out cross-validation framework) plotted against the number of cumulative principal components sequentially introduced to the ridge regression models. The red diamond line represents the top-10 best-predicted regions, while the gray circular line denotes the average across all 360 cortical parcels in the HCP-MMP1.0 atlas. The robust performance increase in semantic hubs from PC 4 onwards reveals that higher-order, abstract semantic dimensions are required to capture higher-order cortical dynamics.

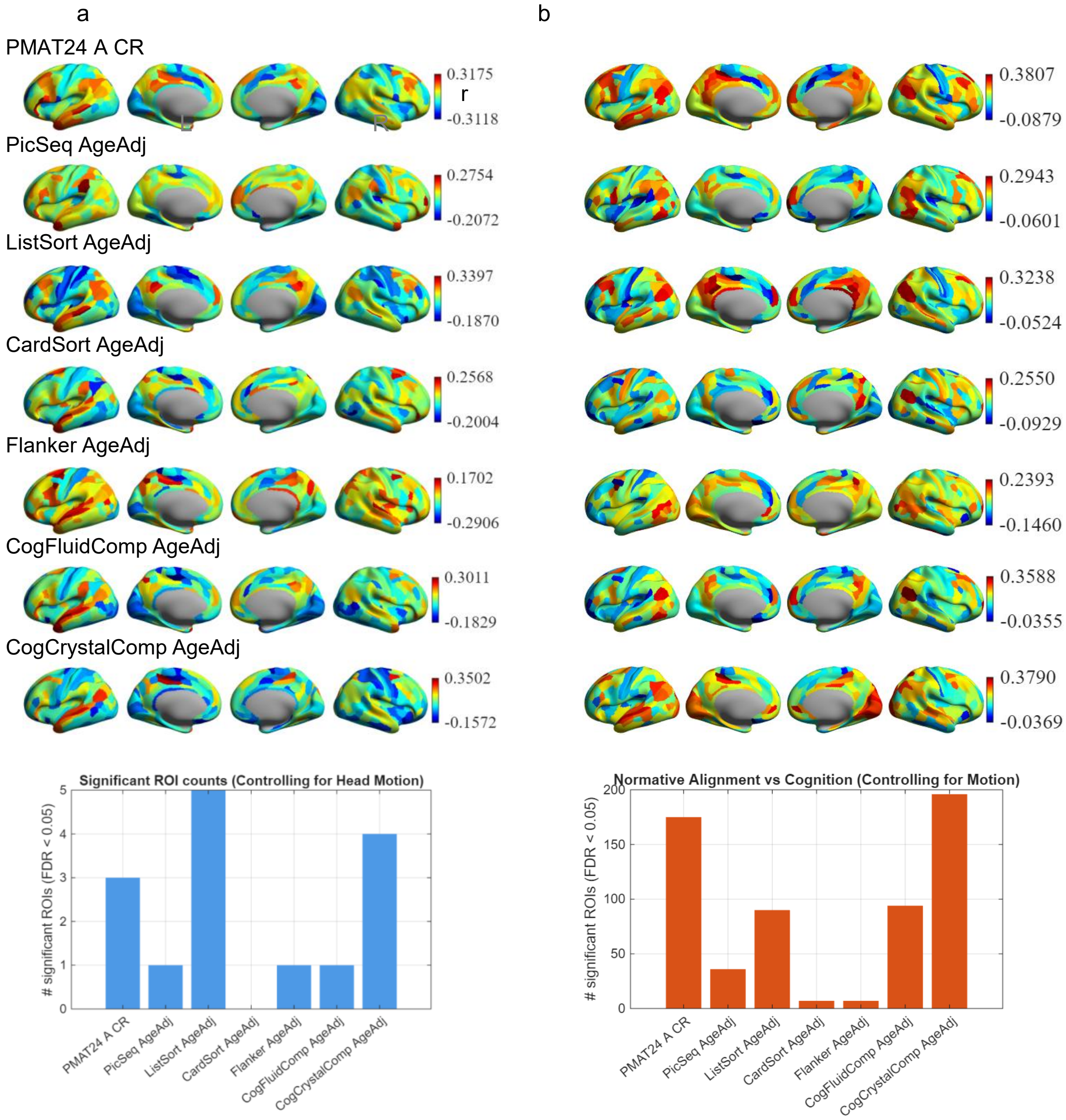

Figure S4. Dissociation between Brain-AI Alignment and Normative Alignment in predicting individual cognitive abilities. All analyses display partial Spearman correlation coefficients (r) controlling for subject mean head motion. (a) AI-Brain Semantic Alignment: Cortical maps and bar plots show the correlation between cognitive scores and the explanatory power of the 11-dimensional AI semantic features (R^2_{AI}). The regression models generating these R^2_{AI} values were trained independently for each subject. The number of significant associations (FDR < 0.05) for each score are shown below. (b) Normative Alignment (Control Analysis): Cortical maps and bar plots show the correlation between cognitive scores and the temporal similarity of BOLD response of each individual to the actual Group Average BOLD response ($R^2_{normative}$). The number of significant associations (FDR < 0.05) for each score are shown below.

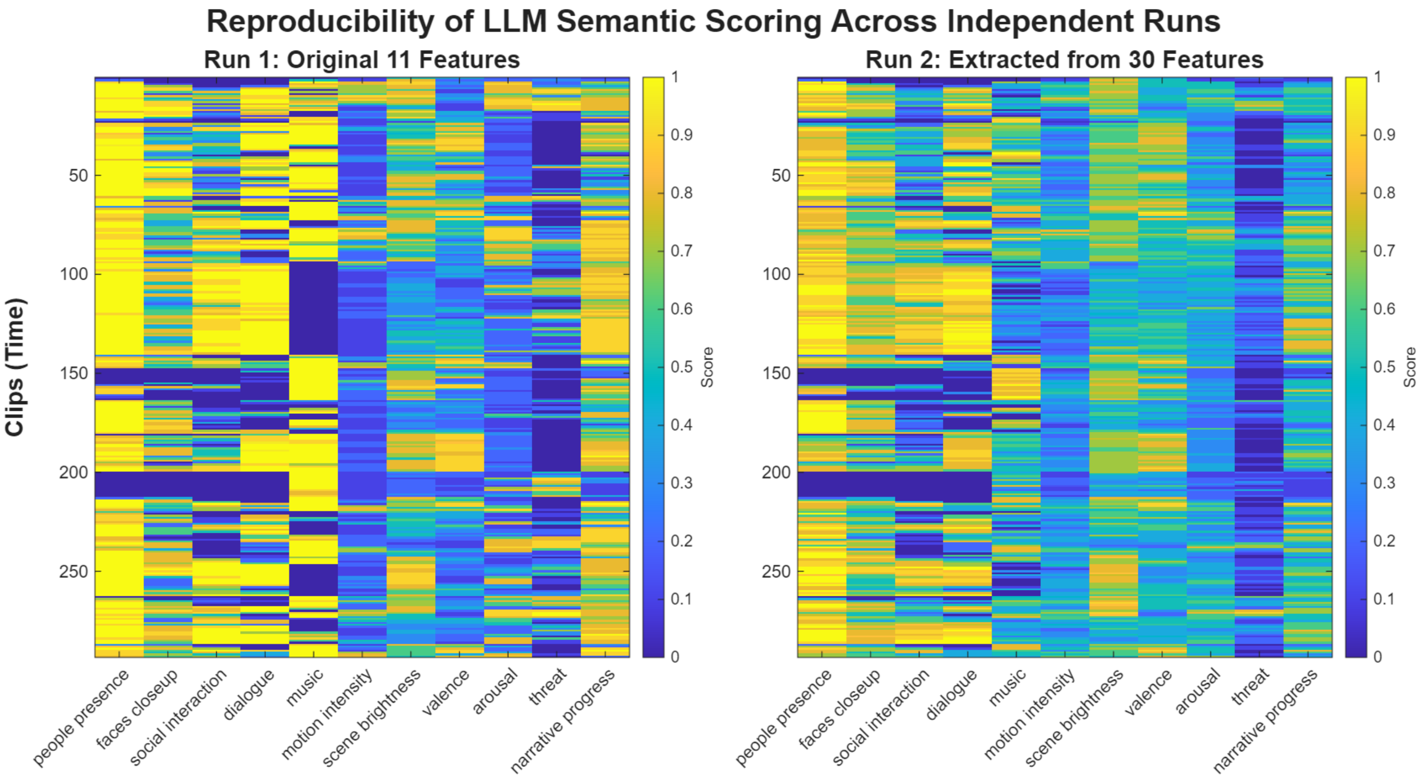

Figure S5. Test-retest reproducibility of the LLM-based semantic annotations. To evaluate the stability of the stochastic language model, we compared the raw semantic scores extracted from two completely independent LLM runs. The second run was performed four months after the initial extraction and utilized a more complex prompting context (extracting 30 features, from which the 11 core features were isolated). (Left) Heatmap of the original 11-feature annotation matrix. (Right) Heatmap of the corresponding 11 features extracted during the independent replication run. The two scoring matrices exhibit striking visual and temporal correspondence across all movie clips. Quantitative analysis confirmed this high consistency, yielding an average Pearson correlation coefficient of r = 0.815 across all features, thereby validating the robustness and scientific reliability of the LLM-derived semantic representations.

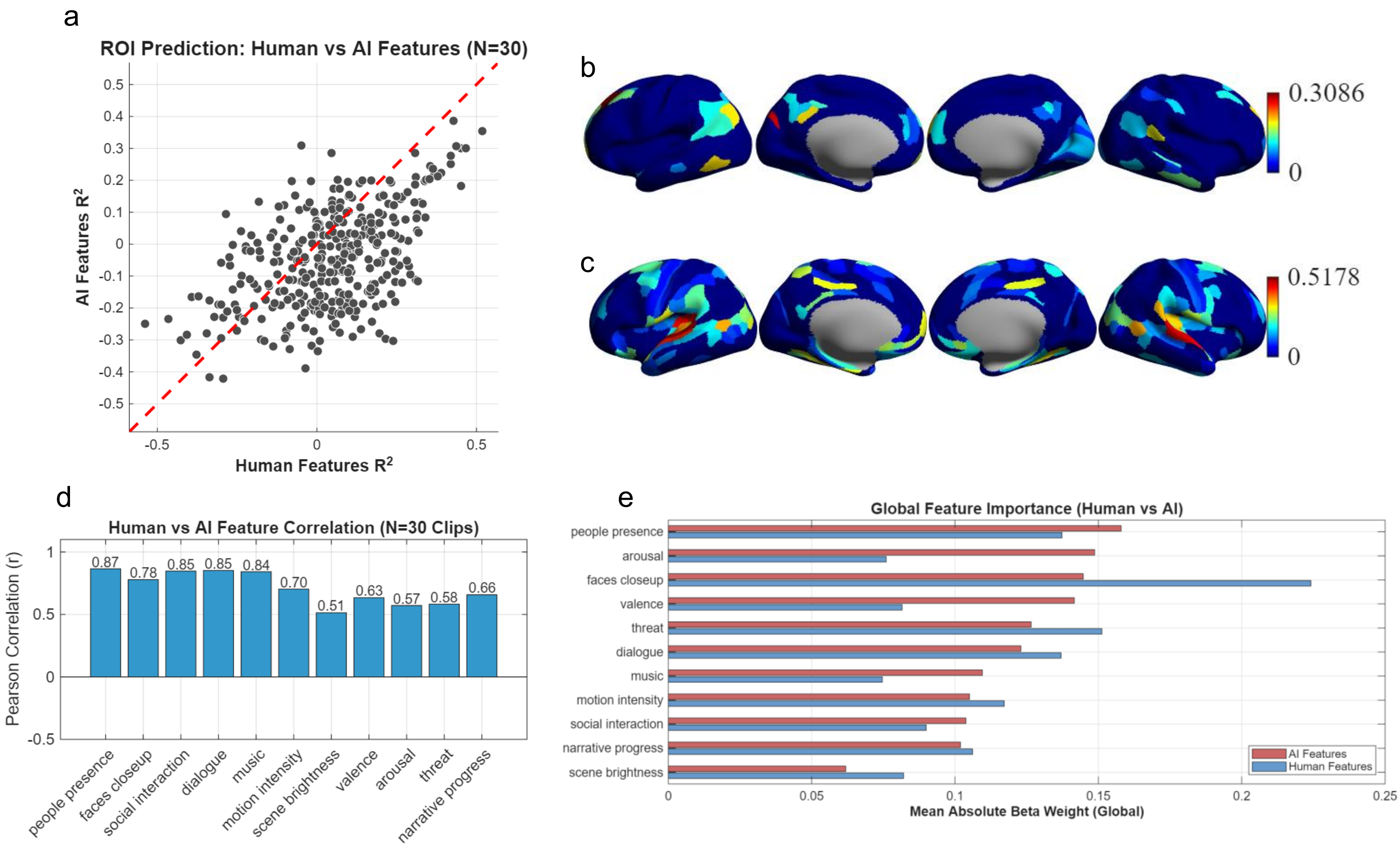

Figure S6. Quantitative comparison between automated AI feature extraction and manual human annotation. To directly evaluate the validity of the LLM-based automated pipeline, an independent subset of 30 video clips was manually annotated by human raters on 11 core perceptual and semantic dimensions. Both feature sets were subsequently evaluated using a Leave-One-Out cross-validation (LOO-CV) Ridge regression framework to predict fMRI responses. (a) ROI-wise comparison of predictive performance. Scatter plot displaying the out-of-sample R^2 for all 360 cortical regions. The x-axis represents the model trained on human-annotated features, while the y-axis represents the model trained on AI-extracted features. The red dashed line denotes the identity line (y = x). (b) Cortical regions with superior AI prediction. Surface projection of out-of-sample R^2 values for brain regions where the AI-derived features outperformed human ratings (thresholded at $R^2 > 0.05$). The AI model demonstrates robust advantages in regions associated with social cognition and higher-order semantic processing. (c) Cortical regions with superior Human prediction. Surface projection of regions where manual human ratings provided superior predictive accuracy (thresholded at $R^2 > 0.05$). (d) Feature-level consistency. Pearson correlation coefficients (r) between manual human ratings and automated AI extractions across the 11 individual features. All features demonstrate strong positive correlations, confirming that the LLM pipeline reliably captures human-like perceptual and cognitive judgments. (e) Global feature importance. Grouped horizontal bar chart comparing the global predictive contribution of each feature for both the AI (red bars) and Human (blue bars) models. Feature importance was quantified by calculating the mean absolute standardized regression coefficients (Beta weights) across all 360 ROIs. The features are sorted by their importance in the AI model in descending order. The analysis reveals a highly consistent macro-level feature weighting strategy between the two models, alongside specific modalities (e.g., people presence, arousal, valence) where the AI-derived features captured additional predictive variance in selected regions relative to human ratings.

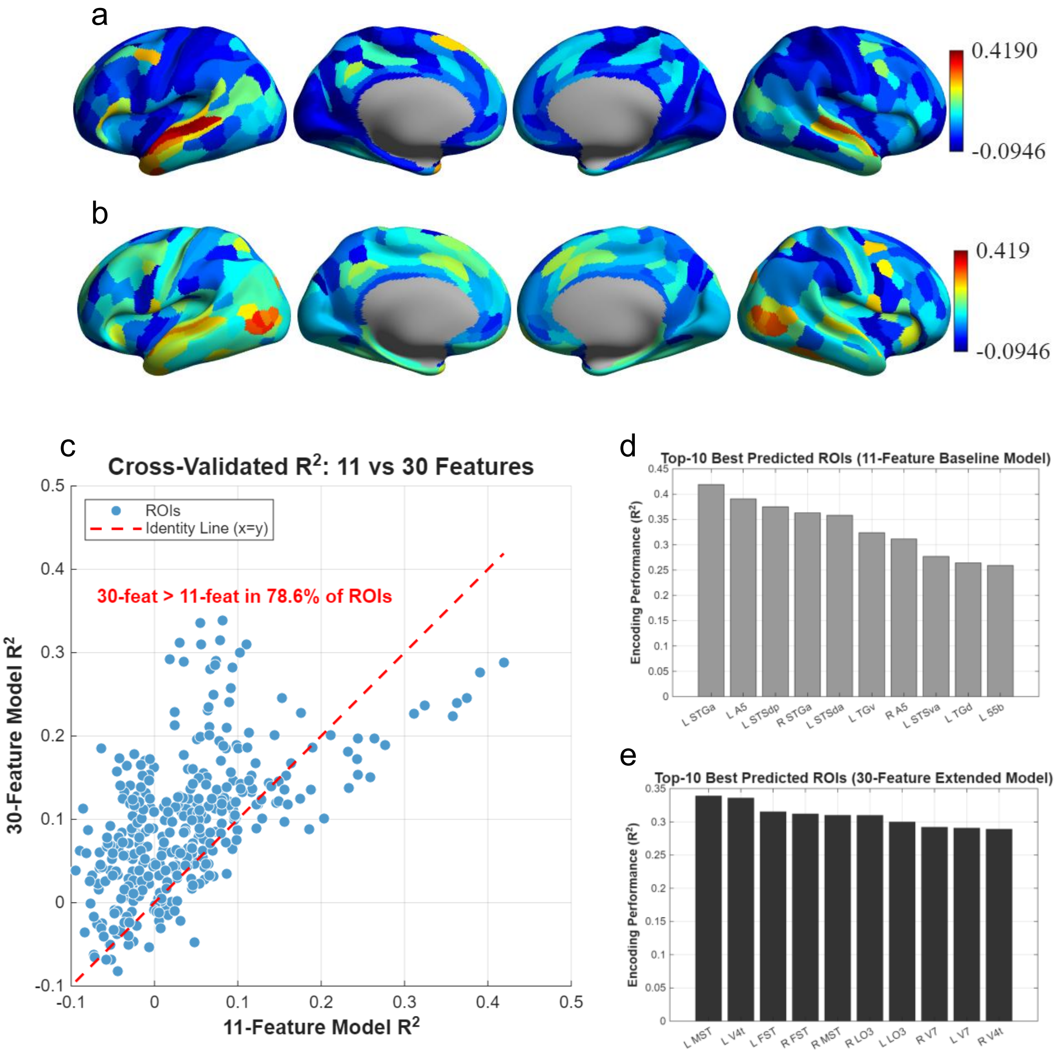

Figure S7. Scalability of the LLM-based semantic annotation framework. To demonstrate the easy expandability of our method, we compared the out-of-sample prediction accuracy (R^2) between the 11 core features and an expanded 30-feature space. (a) Cortical surface map showing the cross-validated R^2 using the original 11 core features (retained in the main text). (b) Cortical surface map utilizing the expanded 30-dimensional semantic space. The extraction of these 30 features was performed entirely independently 4 months after the initial extraction, proving the high reproducibility of the LLM annotation. (c) ROI-wise comparison of out-of-sample predictive power. The scatter plot displays the cross-validated R^2 of all 360 cortical regions of interest (ROIs) for the 11-feature baseline model (x-axis) vs. the 30-feature extended model (y-axis). The red dashed line represents the identity line (x = y). Points above the diagonal indicate superior predictive performance by the 30-feature model, which systematically outperforms the baseline model in 78.6% of all cortical regions. (d) Peak predictive performance of the 11-feature baseline model. The bar chart displays the out-of-sample R^2 values (gray bars) for the top 10 best-predicted ROIs by the 11-feature model. These regions are predominantly localized to early sensory areas, particularly auditory processing hubs (e.g., superior temporal gyrus area STGa, lateral belt area A5, and dorsal posterior superior temporal sulcus STSdp). (e) Peak predictive performance of the 30-feature extended model. The bar chart displays the out-of-sample R^2 values (black bars) for the top 10 best-predicted ROIs by the 30-feature model. In contrast to the baseline, these top-performing regions are predominantly localized to higher-order visual association and motion-processing areas (e.g., medial superior temporal area MST, fundus of the superior temporal area FST, and lateral occipital area LO3), reflecting the enhanced capacity of the extended feature set to capture complex, high-level representations.

The 30 features are:

1. motion_intensity: Overall amount of movement on screen. 2. scene_brightness: Overall luminance of the scene. 3. color_saturation: Vividness of colors. 4. visual_clutter: Spatial complexity or number of objects. 5. loudness: Overall audio volume. 6. pitch_variability: Fluctuation in audio frequencies. 7. indoor_outdoor: Extent to which the scene is outdoors. 8. natural_environment: Presence of nature vs. artificial constructs. 9. text_presence: Presence of readable text or symbols on screen. 10. presence_of_people: Extent to which humans are visible. 11. faces_closeup: Prominence of human faces. 12. presence_of_animals: Visibility of non-human animals. 13. hand_gestures_tools: Prominence of hands manipulating objects or gesturing. 14. dialogue: Prominence of spoken human language. 15. music: Prominence of instrumental or vocal music. 16. social_interaction: Degree of active engagement between two or more characters. 17. eye_contact: Extent to which characters make eye contact with each other or the camera. 18. physical_touch: Amount of interpersonal physical contact. 19. social_hierarchy: Prominence of power dynamics, dominance, or submission between characters. 20. social_conflict: Presence of arguing, fighting, or social tension. 21. valence: The emotional tone of the clip. 22. arousal: The level of emotional or physiological excitement. 23. threat: The presence of physical or psychological danger. 24. joy_amusement: Presence of happiness, laughter, or humor. 25. sadness_melancholy: Presence of sorrow, grief, or depression. 26. disgust_aversion: Presence of gross, repulsive, or morally abhorrent content. 27. narrative_progress: The degree to which the plot or story advances. 28. surprise_unexpectedness: The degree of unexpected events or startling moments. 29. semantic_density: The amount of information or complexity happening simultaneously. 30. theory_of_mind: The extent to which a viewer must infer the hidden thoughts, beliefs, or deceptive intents of the characters.

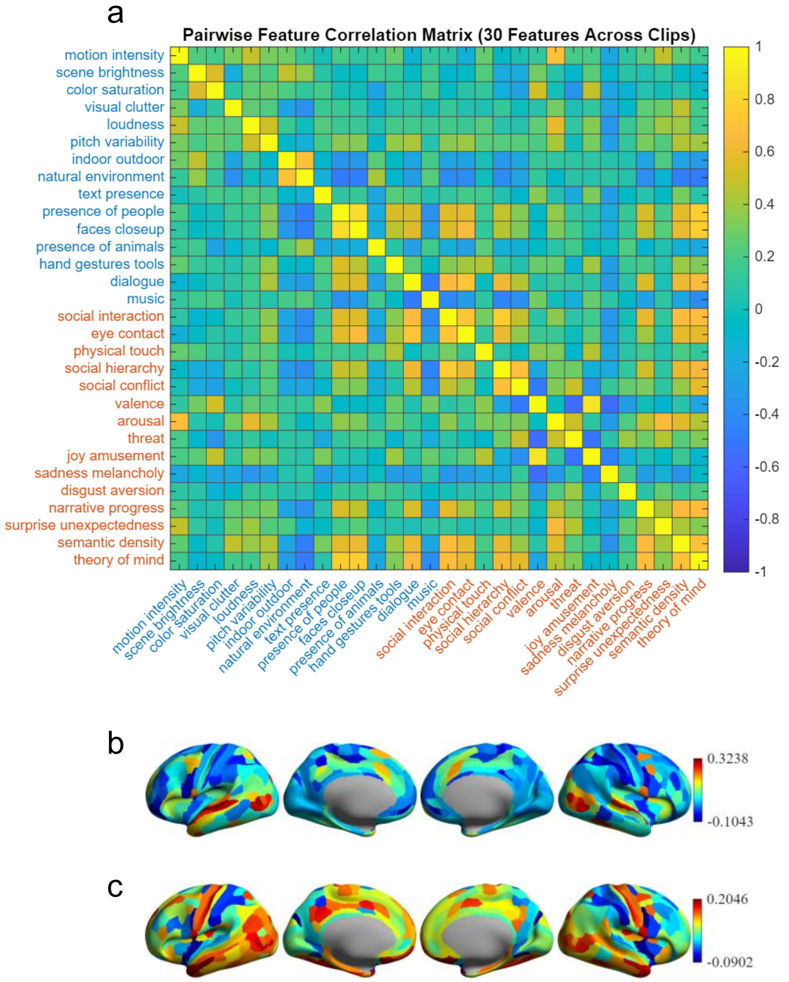

Figure S8. Distinct cortical topographies for encoding low-level sensory statistics and high-level abstract semantics. (a) Pairwise Feature Correlation Matrix. Pearson correlation across all 293 video clips for the expanded 30-dimensional feature space. The features are divided into 15 low-level/concrete audiovisual statistics (Blue names) and 15 LLM-derived high-level abstract cognitive dimensions (Red names). (b) Cortical Encoding Performance of Low-Level Features. Brain surface map displaying the cross-validated R^2 of the baseline model trained exclusively on the 15 low-level sensory features. (c) Cortical Encoding Performance of Abstract Features. Brain surface map displaying the cross-validated prediction accuracy (R^2) of the encoding model trained exclusively on the 15 high-level abstract features.
